# New Epidemiological Aspects of Animal Leishmaniosis in Europe: The Role of Vertebrate Hosts Other Than Dogs

**DOI:** 10.3390/pathogens10030307

**Published:** 2021-03-06

**Authors:** Luís Cardoso, Henk Schallig, Maria Flaminia Persichetti, Maria Grazia Pennisi

**Affiliations:** 1 Department of Veterinary Sciences, and Animal and Veterinary Research Centre (CECAV), University of Trás-os-Montes e Alto Douro (UTAD), 5000-801 Vila Real, Portugal; 2 Department of Medical Microbiology, Experimental Parasitology Section, Amsterdam University Medical Centres, University of Amsterdam, 1105 AZ Amsterdam, The Netherlands; h.d.schallig@amsterdamumc.nl; 3 Department of Veterinary Sciences, University of Messina, Polo Universitario Annunziata, 98168 Messina, Italy; mfpersichetti@gmail.com (M.F.P.); mariagrazia.pennisi@unime.it (M.G.P.)

**Keywords:** birds, disease, epidemiology, Europe, infection, *Leishmania*, leishmaniosis, mammals, reservoir, vertebrates

## Abstract

Infection with *Leishmania* parasites can lead to severe disease in humans and dogs, which act as a reservoir in zoonotic transmission. An increasing number of reports suggest that leishmaniosis is not restricted to dogs, but also affects many other mammalian and avian species. Consequently, this expands the potential reservoir and is of great public and veterinary health concern. The present study reviews, based on a comprehensive search of scientific literature published from 1 January 2001 to 31 December 2020, the currently available information on animal leishmaniosis in vertebrates in Europe, other than dogs and humans. This review provides an exhaustive list of mammals and birds in which infections with or exposure to *Leishmania* parasites have been detected in European countries. Most cases are reported from the Mediterranean region. Domestic animals, in particular cats, pose a concern because of close contact with humans. The wildlife reservoir is less likely to contribute to zoonotic transmission, with the exception of hares. This potentially large reservoir needs to be taken into account when developing control measures for zoonotic leishmaniosis. From a veterinary point of view, it is important that veterinarians are better aware of leishmaniosis and trained in its management.

## 1. Introduction

The parasitic disease leishmaniasis is caused by vector-borne protozoa of the genus *Leishmania* and transmitted via infected female sand flies (*Phlebotomus* and *Lutzomyia*) [1]. The disease is prevalent in tropical and subtropical countries around the world. As a category 1 emerging and uncontrolled disease, leishmaniasis is considered a severely neglected disease and intensified research programs to improve vector control, diagnostics and the therapeutic arsenal to contain further incidence and morbidity are needed [1]. An infection with *Leishmania* parasites in humans can give rise to three main clinical manifestations, depending on infecting *Leishmania* spp. and several host factors, including immune-inflammatory processes [2]. The first is localized cutaneous leishmaniasis (CL) with single to multiple skin ulcers, satellite lesions or nodular lymphangitis [3]. The second is CL with mucosal involvement and the third systemic visceral leishmaniasis (VL) without involvement of the skin and affecting internal organs, such as liver, spleen and bone marrow, which is lethal if not appropriately treated [1,2]. Vector-borne transmission of leishmaniasis to humans can either be anthroponotic (from human to human) or zoonotic (from a non-human vertebrate reservoir to humans) [4]. The identification of the zoonotic reservoirs (i.e., animal populations which may be sources of *Leishmania* spp.) is of great importance to leishmaniasis control programs, in particular to design adequate interventions. In addition, *Leishmania* spp. can also cause considerable disease in animals. In the case of non-human animals, the term leishmaniosis is used to refer to the disease [5].

Dogs are considered to be the main or primary domestic reservoir for human infection in settings where zoonotic leishmaniosis occurs [6]. In fact, a significant proportion of infected dogs remain clinically healthy thanks to an adequate cell-mediated immune response, but these subclinically infected animals can act as carriers of *Leishmania* spp. and are capable of transmitting parasites to the sand fly vectors [7]. However, a variable time after infection, dogs may develop a systemic chronic disease with a broad spectrum of severity and clinical signs due to the dissemination of the parasite in the skin and internal organs and due to the development of immune-mediated pathology. Prognosis can be poor in dogs even when they are treated and chronic kidney disease is its most important determinant [8].

Along with dogs, there are increasing numbers of studies reporting infection of other domestic and wild animals by *Leishmania* spp. This may have important consequences for the development of control programs developed according to a “One Health” perspective, in case of possible transmission to humans from reservoirs that were previously not (well) identified [9]. For instance, in north-eastern Italy, a non-canine reservoir host was suspected for the human cases of VL diagnosed in recent years, based on multilocus microsatellite typing of canine, human and sand fly isolates [10].

Furthermore, increasing awareness of animal leishmaniosis is relevant for veterinary health care providers to ensure that the disease is included in the list of differential diagnoses compatible with a disease state and well managed. Finally, in the case of endangered vertebrate wild species, leishmaniosis could contribute to increase their risk of extinction [11].

The present study reviews the currently available literature on animal leishmaniosis in vertebrates in Europe other than dogs and humans.

## 2. Methods of Literature Search and Review

*Leishmania* in vertebrate hosts other than dogs and humans in Europe is reviewed based on a comprehensive search of the scientific literature published from 01 January 2001 to 31 December 2020 (with data not older than 2001) by primarily sourcing MEDLINE^®^ bibliographic database through PubMed. The following combination of keywords was used/cross referenced: Leish* AND (control OR disease OR epidem* OR host OR infection OR one health OR reservoir OR transmission) AND (animal OR badger OR bat OR cat OR domestic OR donkey OR equine OR feline OR fox OR genet OR hare OR horse OR jackal OR lagomorph OR lynx OR mammal OR marten OR mice OR mouse OR rabbit OR rat OR rodent OR tiger OR wild* OR wolf) AND (Albania OR Andorra OR Armenia OR Austria OR Azerbaijan OR Belarus OR Belgium OR Bosnia Herzegovina OR Bulgaria OR Croatia OR Cyprus OR Czech Republic OR Denmark OR Estonia OR Europe OR Finland OR France OR Georgia OR Germany OR Greece OR Hungary OR Iceland OR Ireland OR Italy OR Kazakhstan OR Latvia OR Liechtenstein OR Lithuania OR Luxembourg OR Macedonia OR Malta OR Moldova OR Monaco OR Montenegro OR Netherlands OR Norway OR Poland OR Portugal OR Romania OR Russia OR San Marino OR Serbia OR Slovakia OR Slovenia OR Spain OR Sweden OR Switzerland OR Turkey OR Ukraine OR United Kingdom OR Vatican).

This review presents a list of mammals and birds in which infections with or exposure to *Leishmania* spp. have been detected in European countries. The most relevant retrieved information per host and parasitic species is presented based on the following covered topics: (a) positivity in tissue smears, sections or cultures; (b) prevalence of leishmanial DNA or parasite-specific antibodies; (c) disease cases and clinical aspects; (d) infectiousness to vector sand flies; and (e) potential reservoir role. Mammalian and then avian species are alphabetically displayed by order, family and species/subspecies found positive. Mention to vertebrate species found negative is also made at the end of their order or family divisions if the species were tested but the results were negative. Data are provided by chronological and geographical order, as much as possible.

## 3. Mammals (Class Mammalia)

### 3.1. Order Artiodactyla

#### 3.1.1. Family Bovidae

##### European Cattle (*Bos taurus*)

In April 2009, a 7-year-old Brown Swiss cow, born and permanently maintained in Switzerland, was found with several ulcerative or plaque-like cutaneous lesions of 1–10 cm of diameter on the muzzle, bilateral carpal regions, ear bases, udder and thoracic wall, and a very large oval-shaped ulcerated cutaneous mass approximately 20 × 5 × 4 cm on the ventral abdomen in front of the udder. Serological testing for antibodies to *Leishmania* yielded positive results both by an immunofluorescence antibody test (IFAT) and an enzyme-linked immunosorbent assay (ELISA), while all other seven cows from the same farm were clearly seronegative [12]. Comparative sequence analysis of polymerase chain reaction (PCR) products showed 98% identity with a presumed new species originally detected in a case of human VL in an immunocompetent patient from southern Thailand [13] and full identity with a leishmanial pathogen described in horses from Switzerland and Germany [14] (see also Horse, under 3.7.1. Family Equidae). The agent, initially referred to as “Leishmania siamensis”, has recently been found to be molecularly identical to *Leishmania martiniquensis* sp. nov. [15] described from the Caribbean island of Martinique and locally responsible for cases of human cutaneous leishmaniosis. The cow was in the 7th month of pregnancy and after delivery the owner reported spontaneous improvement of the skin lesions. The novel *Leishmania* sp. described in Central Europe sporadically causing cutaneous leishmaniosis in horses [14] seems to have extended its host range to cattle. The potential transmitting vectors for all these cases have not yet been identified [12]. Twelve additional clinical cases suspected of leishmaniosis in bovines from Switzerland were all negative for *Leishmania* [16].

##### Domestic Goat (*Capra aegagrus* Hircus)

In Thessaly, central Greece, a total of 179 goat serum samples collected from 20 farms were all negative for antibodies to *Leishmania* by ELISA [17].

##### Domestic Sheep (*Ovis aries*)

Additionally, in Thessaly, of 361 sheep sera collected from 34 farms, 0% were found positive for antibodies to *Leishmania* by ELISA [17].

### 3.2. Order Carnivora

#### 3.2.1. Family Canidae

##### Golden Jackal (*Canis aureus*)

In 12 sites all over Serbia, 15 (6.9%) of 216 spleen samples collected from jackals during a 26 month period (2010–2013) were positive for *Leishmania* by PCR [18].

In Georgia, one (2.6%) of 39 jackals was found positive for antibodies to *Leishmania* by a commercial rK39 rapid diagnostic test [19]. Jackals and foxes (see also Red Fox) are not considered the main source of *Leishmania* in Georgia.

In Romania, out of 54 jackals legally hunted between October 2013 and May 2015, bone marrow of one single animal (1.9%) from the southern Dolj County was found positive for *L. infantum* by PCR and DNA sequencing [20].

##### Gray Wolf (*Canis lupus*)

One case of *L. infantum* infection was reported in a 4-year-old gray wolf from Croatia found dead, in November 2003, in central-southern Dalmatia, where canine leishmaniosis (CanL) is endemic. Observed pathological manifestations included chronic dermatitis, generalized hair loss, white scaling, skin erosions and ulcerations, cachexia, orchitis, lymphadenomegaly and hepatosplenomegaly, which are coincident with those of leishmaniosis described in dogs. Amastigotes were detected in macrophages of peripheral blood, lymph nodes, spleen, liver, skin, adrenal glands, and testes [21].

A positive but low level of *L. infantum* DNA was detected by real-time quantitative PCR (qPCR) in the peripheral blood of one captive wolf from the European Breeding of Endangered Species Programme (EEP) from Lisbon (Portugal) that had been relocated from Lleida (north-eastern Spain). It is not known where this wolf became infected, as it had been exchanged between institutions before the infection was discovered. Surveillance of *Leishmania* infection in the captive lupine population of Europe is recommended for zoonotic and conservation issues, especially in areas where CanL is endemic [22].

Two (4.1%) of 49 wolves from north-western Spain (communities of Asturias and Galicia) had *Leishmania* kinetoplastid DNA (kDNA) in the hair of their ears, as detected by real-time PCR [23].

In 88 wild wolves from the community of Asturias, which were submitted for necropsy between 2004 and 2010, the seroprevalence of antibodies to *Leishmania* was 0% by ELISA. However, in a subset of 39 wolves sampled from 2008 to 2010, 46.2% animals were molecularly positive for *Leishmania* by PCR on DNA extracted from mandibular lymph nodes [24]. This value is higher than the 18.1% molecular prevalence, by PCR from spleen and blood, in wolves obtained between 1997 and 2007 also in Asturias by Sobrino et al. [25]. The seronegative status was attributed to a potential lack of immune response by the apparently healthy wolves found to be molecularly positive for *Leishmania* [24].

Still in Asturias, 102 wolves were necropsied between 2008 and 2014, as part of an established wildlife sanitary surveillance program, with the causes of death comprising population control hunts carried out by wildlife officers (*n* = 85), vehicle collisions (*n* = 13) and non-determined ones (*n* = 4). A 33.3% molecular prevalence of *Leishmania* infection was detected by means of PCR on spleen samples, with the identification of *L. infantum* being confirmed by DNA sequencing of positive samples. Histological analyses revealed that macroscopic cutaneous lesions observed in several wolves were associated with mange, while no other leishmaniosis-compatible lesions were detected. A widespread positivity to *Leishmania* and an apparent increase in prevalence during the years of 2004–2014 were observed in wolves from Asturias. Although these parasites do not seem to represent a threat to the maintenance of Asturian lupine populations, wolves may be useful sentinel species for the detection of *L. infantum* in the field [26].

In south-eastern Spain, between 2013 and 2015, one (33.3%) of three wolves which belonged to a zoological park was found real-time PCR positive to *Leishmania* in a skin sample [27].

One (50%) of two wolves from a zoological park in Murcia, south-eastern Spain, sampled in the period 2008–2017, was positive in spleen, liver or skin by a real-time PCR targeting the ITS2 of *L. infantum*. Analysis of kDNA sequences revealed single-nucleotide polymorphism (SNP)-derived genotype 2 in one wolf, as well as in domestic dogs [28].

Eight (25%) of 32 wolves collected after road kills in the region of Piedmont, north-western Italy, between 2009 and 2017, were positive for *Leishmania* by a conventional PCR on spleen samples. DNA sequences showed 100% identity with *L. infantum* [29].

##### Red Fox (*Vulpes vulpes*)

In the western part of the Campania region, southern Italy, *Leishmania* was detected by PCR in 20 (40%) of 50 fox carcasses collected from May 2004 to May 2005. All of those 20 foxes were PCR positive simultaneously in popliteal lymph node and bone marrow samples, whereas just 17 cases were also PCR positive in skin samples. Infection status was not associated with sex or age (mature versus young animals) [30].

Additionally, in Campania, in the Picentini hills, 10 (20.8%) of 48 foxes shot by hunters, from October 2012 to January 2013, or killed in road accidents, between June and September 2013, were found infected with *Leishmania* by nested-PCR (nPCR). The overall n-PCR positivity was 17.4% for spleen and 13.3% for lymph nodes, with four animals positive in both samples; all liver samples were negative. Additional PCR and restriction fragment length polymorphism (RFLP) analysis confirmed *L. infantum* as the infecting species. There were no statistically significant association between *Leishmania* infection and sex, age or the presence of lung or intestinal endoparasites. In the PCR-positive foxes, no macroscopic alterations suggestive of leishmaniosis were recorded at necropsy [31]. Seroprevalence in 123 fox-hunting dogs sampled from April to September 2012 was 17.9%, as revealed by IFAT, and the risk of exposure in red foxes was assumed to be lower than in the hunting dogs living in the studied area [31].

In the province of Pisa, central Italy, 92 adult foxes, shot during the regular hunting seasons from January 2008 to July 2009, were examined by Verin et al. [32]. None of the animals presented lesions compatible with leishmaniosis. Forty-eight foxes (52.2%) had leishmanial DNA in lymph nodes, including eight (8.7%) positive in spleen, but skin samples were PCR negative. A second PCR with species-specific primers confirmed the presence of *L. infantum* in all previously positive samples. The distribution of parasitic DNA between mature (41/67, 61.2%) and young (7/25, 28%) foxes was statistically different, but not between males (30/60, 50%) and females (18/32, 56.2%). Furthermore, all sera were scored negative by IFAT for specific antibodies to *Leishmania*. The significant difference between young and mature foxes could be explained by a greater likelihood of contact with infected sand flies during the life span of these mammals [32].

Two (28.6%) of seven red foxes found dead on the road in the province of Palermo, Sicily, from 2015 to 2017, tested positive in skin, spleen and popliteal lymph node or in spleen only for *L. infantum* kDNA by qPCR. None of the *Leishmania*-infected animals showed macroscopic alterations at necropsy [33].

Nineteen (12.3%) of 155 red foxes officially hunted in the region of Piedmont, north-western Italy, between 2009 and 2017, were positive for *Leishmania* by a conventional PCR on spleen samples. DNA sequences showed 100% identity with *L. infantum* [29].

Six (66.7%) of nine foxes from the community of Extremadura, western Spain, had *Leishmania* kDNA in the hair of their ears or legs, as detected by real-time PCR [23].

In the provinces of Biscay, Gipuzkoa and Álava, Spanish Basque Country, *Leishmania* kDNA was detected by real-time PCR in macerates of liver and spleen from 14 (29.2%) of 48 foxes hunted or found dead between 2001 and 2006. A subsequent conventional PCR amplifying the internal transcribed spacer (ITS) 2 and DNA sequencing confirmed *L. infantum* [34].

*Leishmania infantum* zimodeme MON-1 was detected by optical microscopy of blood smears, culture and enzymatic characterisation in 48 foxes (12%) of 400 from the province of Soria, northern Spain. Infection was more prevalent in juveniles (24.6%, 15/61) than in adults (8.6%, 20/233) or old foxes (12.3%, 13/106). Of the infected animals, 2.5% had enlarged livers and spleens, and two other showed cutaneous manifestations compatible with leishmaniosis [35].

In Asturias, north-western Spain, *L. infantum* was detected by PCR and DNA sequencing in spleen samples from six (46.2%) of 13 red foxes collected from January 2008 to May 2014 [26].

In the region of Murcia and the Valencian community, south-eastern Spain, 31 (44.9%) of 69 foxes collected between 2013 and 2015 were found positive by real-time PCR in tissue samples including skin, spleen or liver. Foxes were much more likely to be positive in organs other than skin. Lesions compatible with leishmaniosis were not observed in any of the animals. There was some evidence of a seasonal pattern in *L. infantum* prevalence in foxes and it was lower in spring compared to other seasons. Analysis of kDNA by PCR-RFLP revealed *L. infantum* B genotype in all investigated foxes, which is frequent in people and dogs in the Iberian Peninsula and Morocco [27].

Nineteen (73.1%) of 26 foxes from the region of Murcia and the neighbouring province of Alicante, sampled in the period 2008–2017, were positive in spleen, liver or skin by a real-time PCR targeting the ITS2 of *L. infantum*. Analysis of kDNA sequences revealed SNP-derived genotype 2 in foxes, as well as in domestic dogs [28].

In the Var area, south-eastern France, from 2006 to 2012, among 92 red foxes screened by quantitative PCR, prevalence of *L. infantum* infection was 8.7%. DNA was found in spleen (7/92) and liver (2/38), but not kidney (0/23), skin (0/14) or blood (0/15). No suspected leishmaniosis lesions were observed at post-mortem examination [36].

Fourteen (15.1%) of 93 red foxes from the departments of Bouches-du-Rhône and Var, south-eastern France, hunted in the period 2008–2018, were found positive in spleen samples by qPCR for leishmanial kDNA and conventional PCR targeting ITS1. The foxes were from the military camps of Carpiagne (*n* = 57) and Canjuers (*n* = 33) and from the city of Hyères (*n* = 3); 52 were males and 41 females. ITS1 sequences had 99–100% identity with *L. infantum* [37].

*Leishmania infantum* was detected by PCR and sequencing in both blood and bone marrow from one (1.3%) of 78 red foxes obtained in eight districts of northern, central and southern Portugal, between November 2008 and March 2010. The positive animal was from the northern district of Vila Real and seemed not to suffer from gross lesions or histological abnormalities due to leishmanial infection [38].

In Fthiotida, central Greece, *L. infantum* DNA was detected in spleen, lymph nodes, bone marrow and/or blood samples of 28 (59.6%) of 47 red foxes found dead or captured, narcotized and freed after bleeding, from November 2009 to 2011. PCR positivity was not associated with animal age, sex, health status or presence of endo- and ectoparasites. These results were coincident with the seroprevalence found in dogs in the area. Three of nine vulpine sera tested by IFAT had IgG antibodies to *Leishmania*. RFLP analysis revealed the presence of *L. infantum* in the PCR-positive animals. There was a significantly higher number of PCR-positive animals living at <300 m altitude compared with animals living at higher altitudes. There was no statistical relationship of gender, age, the presence of ecto- or endoparasites or health status of the animal and PCR positivity [39].

In Georgia, one (2.6%) of 38 foxes sampled during the year 2012 was seropositive for *Leishmania* by a commercial rK39 rapid test [19] (see also Golden Jackal).

#### 3.2.2. Family Felidae

##### Domestic Cat (*Felis* Catus)

An increasing number of studies have been conducted in endemic areas all around the world to investigate *Leishmania* infection in cats. At the same time, case reports of FeL caused by *L. infantum* were described over the last 30 years. Most of them are not available in MEDLINE bibliographic database and will not be included in this review, but they were reviewed in 2015 and 2018 by Pennisi et al. [40] and Pennisi and Persichetti [41], respectively.

*Leishmania infantum* (or *Leishmania donovani* according to the recognition of *L. donovani* complex as a single species) is the species almost exclusively detected in cats in the endemic areas of several Mediterranean countries (Italy, Spain, Portugal, France, Greece, Turkey, Cyprus) [40]. However, DNA of *Leishmania major* was detected in cats from Turkey and Lisbon (Portugal) and of *Leishmania tropica* from Turkey [42,43,44].

*Leishmania infantum* parasites detected in cats share the same genetic characteristics with *L. infantum* strains isolated from humans, dogs, and sand flies [44,45]. Many data about parasitological and immunological positivity to *L. infantum* of cats from European endemic areas are available and they are summarized in Table 1. These studies are extremely heterogeneous based on the characteristics of the population tested, the techniques and methodologies (particularly the cut-off values) used, but they all suggest that cats are frequently exposed to *L. infantum* infection. As seen in dogs, *Leishmania* PCR-positive healthy cats can be negative for anti-*Leishmania* antibodies [40,46]. Cell-mediated adaptive immune response elicited by *L. infantum* in exposed cats from endemic areas was investigated in one single study and specific IFN-γ was detected in 21% of tested cats and they all were blood PCR negative and negative or borderline antibody positive [47]. However, the relationship between the pattern of adaptive immune response and the severity of feline leishmaniosis (FeL) has not yet been investigated. The long-term duration of feline infections has been substantiated by investigations performed out of the transmission season and confirmed by longitudinal evaluations [48,49,50].

However, when prevalence or annual incidence in dog and cat populations are compared, feline antibody and PCR positivity are found lower [64,71].

An overall number of 53 clinical cases of FeL were detected by the bibliographic search conducted in the present review and the vast amount of information available is summarized below.

In 2004, a series of four cases diagnosed in the Messina province (Sicily, Italy) between December 1997 and May 2001 were reported [89]. Case 1 was a 14-year-old, female, domestic short-haired (DSH) cat admitted for dyspnoea, lethargy, anorexia and weight loss. Pale mucous membranes, dehydration, dermatologic lesions on the head (a small crusty ulcer and a hemorrhagic bulla), and uveitis sequela were observed at physical examination. Amastigotes were detected at cytological evaluation of skin lesions and the cat was antibody positive to *L. infantum* (IFAT titre: 640). Additional investigations detected feline immunodeficiency virus (FIV) antibody positivity, azotemic chronic kidney disease (CKD), pancytopenia, hyperproteinemia and hyperglobulinemia with monoclonal hypergammaglobulinemia, bronchopneumonia and pyothorax, and the cat died 35 days later. The age of case 2 was unknown and it was a rescued adult long-haired cat admitted for systemic lymph node enlargement. Uveitis sequela was observed at ophthalmologic examination. Complete blood count, biochemical profile and urinalysis were within reference range apart from hyperproteinemia and hyperglobulinemia with monoclonal hypergammaglobulinemia and the cat was FIV antibody and feline leukaemia virus (FeLV) antigen negative. Amastigotes were seen at cytological evaluation of lymph nodes and the diagnosis was confirmed by antibody detection (IFAT titre: 640), positive culture and conventional PCR from lymph nodes. Anti-*Leishmania* oral treatment was started with fluconazole (5 mg/kg q24 h for 2 months) and it was ineffective as well as the subsequent combination of metronidazole (25 mg/kg) and spiramycin (150,000 IU/kg) q24 h for 35 days and afterwards itraconazole (50 mg q24 h for 2 months). After that no anti-*Leishmania* treatment was given. Chronic kidney disease was evidenced 5 months after the diagnosis of leishmaniosis and it progressively worsened associated with severe weight loss and non-regenerative anemia. Twenty-two months later, the diagnosis euthanasia was performed and, at that time, lymph node enlargement was no longer detected, hypergammaglobulinemia was reduced but alfa-globulin fraction was increased and anti-*L. infantum* IFAT titre was 320. Case 3 was a 6-year-old, DSH, male cat presented with a single enlarged lymph node. Conventional PCR and culture from the enlarged lymph node were both positive and anti-*L. infantum* IFAT titre was 1280. A cutaneous hemorrhagic bulla was observed on the pinna margin as well as chorioretinitis. The cat was positive for anti-FIV antibodies and hyperproteinemia, hyperglobulinemia and monoclonal hypergammaglobulinemia were detected. Complete blood count, biochemical profile and urinalysis were in the reference range and the owner did not comply with therapy. Three years later urinary abnormalities suggestive of CKD were found and 5 years after the diagnosis of leishmaniosis the owner reported anorexia and episodes of seizure. A cardiac arrhythmia was diagnosed but the owner refused further investigations and asked for euthanasia. The initial clinical findings persisted during the 5 year period of follow up and, at the time of euthanasia, anti-*L. infantum* IFAT titre was 640 and lymph node was found *Leishmania* positive at cytological and PCR investigations. Case 4 was a 10-year-old, DSH, female cat admitted for lethargy, anorexia and severe weight loss. Pale mucous membranes, systemic lymph node enlargement, alopecia, hepatomegaly and acute uveitis were observed at physical examination. The cat was found positive for anti-FIV antibodies and pancytopenia, hyperproteinemia, hyperglobulinemia and monoclonal hypergammaglobulinemia were detected. Lymph nodes were found positive to *L. infantum* by cytological evaluation, PCR and culture. Anti-*Leishmania* antibodies were detected by IFAT (titre: 640). The cat was treated with allopurinol (20 mg/kg q24 h) and erythropoietin was added during the first two months and human recombinant interferon-alpha was given for 6 months. In a few weeks there was a clinical improvement and in few months was clinically cured. Allopurinol was stopped after 15 months of therapy when anti-*Leishmania* antibodies were <80 and PCR was negative. However, a few months later, there was a clinical recurrence associated with antibody positivity (IFAT titre: 1280) and positive culture from lymph node. These four cats were tested, apart for FIV and FeLV infections, also for antibodies against feline coronavirus (FCoV), *Bartonella henselae* and *Toxoplasma gondii*. Anti-FCoV antibodies were detected only in case 2 but with a low IFAT titre (25). Case 2 and case 3 were both IgG positive for *B. henselae* (IFAT titre: 256). Case 1 (titre: 100), case 2 (titre: 400) and case 4 (titre: 6400) were found IgG positive for *T. gondii* [89].

In 2001, a pseudotumoral ocular mass (approximately 1.5 cm in diameter) was seen in a 6-year-old, female, European cat from Spain affected by monolateral uveitis [90]. The cat was antibody positive to feline immunodeficiency virus (FIV) and slight lymph node enlargement was found as well as stomatitis and faucitis. After enucleation, a severe granulomatous inflammation of choroid layer, iris and ciliary process were found with macrophagic cells containing numerous amastigotes and similarly in the Descemet’s membrane. Ultrastructural study for the first time documented ocular lesions caused by *Leishmania* spp. in a cat but unfortunately IHC or parasite DNA detection were not evaluated [90].

A case was diagnosed in Imperia (Liguria, Italy) in 2002 in a 6-year-old, female, European cat admitted with a history of lethargy and an ulcerated nodule (2 cm in diameter) on the eyelid [91]. Clinical examination revealed weight loss, reduced appetite, severe ulcerative stomatitis, generalized lymphoadenopathy and splenomegaly. Routine clinicopathological evaluation reported a neutrophilic leukocytosis, and the cat was FIV antibody positive and antigen positive to FeLV. *Leishmania* amastigotes were found at cytological evaluation of the palpebral nodule and of a popliteal lymph node. The palpebral nodule was surgically removed and a pyogranulomatous dermatitis extending to the ulcerated eyelid mucosa was found with many amastigotes in almost all macrophages. Cutaneous adnexa were also involved and epidermis was affected by hyperplasia and hyperkeratosis with focal crusts containing macrophages filled by amastigotes. *Leishmania* amastigote ultrastructure was recognized at electron microscopy evaluation of the nodule and positivity of amastigotes was seen by IHC investigation. Samples from the nodule and lymph nodes were inoculated into Syrian hamsters and Evans’ modified Tobie’s medium. Promastigotes were observed after 7–10 days, but the strain was not isolated and xenodiagnosis was negative. Cytological smears were also used to perform PCR investigation and *L. infantum* was identified by PCR-RFLP analysis. The cat was antibody positive to *L. infantum* (IFAT titre: 80) [91].

Leishmaniosis was diagnosed (first diagnosis probably in 2004) in a 6-year-old DSH spayed female cat from the Var region in the south of France [92]. The cat was affected by uveitis that evolved to panuveitis with hydrophthalmus affecting both eyes approximately 1 year apart and, after enucleation, a pseudotumoral granulomatous uveitis was diagnosed with amastigotes seen in numerous histiocytes. Serological investigations were negative for FIV, FeLV and feline infectious peritonitis virus (FIPV) infections, but observed a low positive IFAT titre for *Toxoplasma* (40) and a high positive IFAT titre for *Leishmania* (800). The affected ocular tissue was *Leishmania* PCR positive (400,000 amastigotes/10^6^ cells). Interestingly, no anti-leishmanial treatment was given and 4 years later, the cat was seen for a proliferative gingival lesions and a *Leishmania*-associated granulomatous gingivitis was diagnosed by histopathology [92].

Navarro et al. [78] reported the prevalence of *Leishmania* infection out of 2632 feline skin and ocular biopsies examined over the 4 year period 2004–2007 (Table 1). Limited clinical details were available about the 15 cats found positive at histological examination, confirmed by IHC. The breed was known for 15 cats (12 European and two Siamese breed), the age for 10 cats (range 2–14 years), and the sex for 13 (6 males and 7 females). Seven positive skin biopsies included four cases with multiple cutaneous nodules (from 1 cat dead for CKD associated with leishmaniosis, the liver, spleen and kidney tissues were also examined), one forelimb ulceration, one pododermatitis and one exfoliative dermatitis. The five eye tissues concerned two cats with panophthalmitis, two with conjunctivitis and one with blepharitis. This latter case and one with conjunctivitis were clinically cured with allopurinol treatment. The three cats with mucocutaneous lesions had nodules on the lips (2 cats but 1 of them also on the ear, eye and anus), or rhinitis (one cat) and two of them were died for CKD few months after diagnosis. The most common histopathological feature observed in all examined tissues was a diffuse granulomatous inflammation, with macrophages containing numerous amastigotes (apart from the kidney) and variable numbers of lymphocytes and plasma cells. However, granulomatous perifolliculitis was seen in a nodular lesion and lichenoid interface dermatitis in the exfoliative dermatitis, and in these cases fewer amastigotes were found [78].

Another clinical case diagnosed in the south of France (Grasse city area) was published in 2005 [93]. A 13-year-old, DSH, spayed female cat was presented to veterinary consultation because of an ulceration on the left temporal region persisting since 2 months. General condition was good and a diagnosis of squamous cell carcinoma associated with *Leishmania* infection was based on histopathology of biopsied skin lesion. *Leishmania* infection was confirmed also by antibody detection (IFAT titre: 800), ELISA and Western blot (WB). Interestingly, blood culture was positive and *L. infantum*, zymodeme MON-1 was identified. Concurrent FIV and FeLV infections were diagnosed by detection of FIV antibodies and FeLV p27 antigen, and euthanasia was required by the owner. At necropsy, amastigotes were seen in the spleen and lymph nodes [93].

Rüfenacht et al. [94] reported in Switzerland (a non-endemic country) two cases of leishmaniosis diagnosed respectively in a cat imported from Spain (Mallorca) and in a cat that spent some time in this endemic country. The cat from Mallorca island was a 6-year-old, neutered male, Siamese cross stray cat. Since importation (1 year before diagnosis) the cat showed a single ulcerated nodule in the left tarsal region but an additional similar lesion on the thorax had disappeared spontaneously in the meantime. The nodule was totally excised and a diffuse infiltration with macrophages containing many intracellular amastigotes, plasma cells and some neutrophils were observed. *Leishmania* infection was confirmed by PCR from the skin. A tarsal ulcerated plaque recurred shortly after excision and no other abnormalities were observed except for marked hyperglobulinaemia and slight hypoalbuminaemia. Serological testing for FIV and FeLV were negative and *Leishmania* amastigotes were detected by cytological examination of bone marrow. Allopurinol (10 mg/kg q12 h per os [PO]) was started but 3 months later, the lesion had deteriorated and a new skin lesion (hairless plaque) had appeared on the right tarsus. The primary lesion was removed surgically but amastigotes were no longer found histologically. Due to increases in liver enzymes, allopurinol was reduced to 5 mg/kg and cefalexin (20 mg/kg) was given orally q12 h for 10 days. Three months later both skin lesions had healed and hyperglobulinemia improved. Ten months after surgery the cat was healthy, without skin lesions and the owner stopped allopurinol therapy 2 months later. Forty months after the surgery and, finally, in Autumn 2004, this cat was still healthy and had no recurrence of the skin lesions. The cat that lived in Spain was a 9-year-old, DSH, female cat with a 3 year history of recurrent crusts on ear tips, inner surface of the pinna, and neck, pustules and depigmentation of the nose, and mild pruritus. Repeatedly tested for FIV and FeLV, it was always found negative. Additionally, the cat was lethargic and with reduced appetite. Several treatments had been given and transient improvement had been obtained only during oral administration of prednisolone. Pemphigus foliaceus was diagnosed based on cytological and histological examinations of multiple skin lesions. Anti-*Leishmania* antibody detection was strongly positive by ELISA as well as PCR on skin biopsy. However, leishmanial IHC was negative. Mild hyperglobulinemia and leukopenia were found. Allopurinol (10 mg/kg PO q12 h) associated to prednisolone (1 mg/kg PO q48 h for 1 month and q72 h for 1 month) were given and after 10 weeks the cat was healthy, but corticosteroid was maintained once-weekly for 6 months after diagnosis. Two years later, the cat was still healthy and a topically treatment with cortisone was used sporadically when reappearing crusts on the pinnae [94].

In June 2005, leishmaniosis was diagnosed in a 13-year-old, neutered male, DSH cat from Lipari (Sicily, Italy) and for the first time sand flies were infected by feeding on this naturally infected cat [95]. The cat had been several times tested for *Leishmania* infection since 1999, and low positive IFAT titres were sometimes detected and in one instance blood PCR tested positive. Mild periodontitis and enlarged mandibular lymph nodes were the clinical signs observed in 1999. Due to severe stomatitis the cat was given short courses of low dose methylprednisolone and cytarabine from October 2001 to May 2005, when it was referred for stomatitis and systemic lymph node enlargement. The cat was FIV and FeLV negative and anti-*Leishmania* antibody titre was 160. Culture of popliteal lymph node aspirate was performed and tested positive for *Leishmania* promastigotes. The cat was submitted to xenodiagnosis using 4–6-day-old sugar fed sand fly females (*n* = 100) from a laboratory colony of *Phlebotomus perniciosus*. After 90 min exposure in a fine net cage, 20/100 females were blood fed and 4/19 dissected insects were positive for motile promastigotes in the anterior cardia area (21%). The isolate obtained from infected sand flies was identified at isoenzyme analysis as MON-1 zymodeme as the isolate previously obtained from the cat lymph node. Similarly, both strains shared the same RFLP patterns of intergenic and intragenic *cpb* sequences common to strains of the *L. donovani* complex and European *L. infantum* strains [95].

On December 2008, feline leishmaniosis was diagnosed in a 17-year-old, DSH, neutered male living in the south of France (Saint-André-de-la-Roche) [96]. Three years before, the cat had been treated with feline interferon-omega for recurrent pododermatitis and FIV infection, and it was admitted because of weight loss, reduced appetite and lethargy. Additionally, since approximately 1 month, pruritus and skin lesions were observed. The margin of the left pinna was severely ulcerated and an invasive squamous cell carcinoma was histologically diagnosed after surgical resection. Three small papular and ulcerated areas with crusts and scales respectively on the left cheek, inner surface of the pinna and shoulder were biopsied. *Leishmania* amastigotes were seen in macrophages associated with perivasal dermal inflammation and *L. infantum* zymodeme MON-1 was cultured and identified. The cat was antibody positive by WB (14, 18, 21 and 31 kDa bands) and ethylenediamine tetraacetic acid (EDTA) blood PCR was also positive (26 amastigotes/mL). Treatment with allopurinol (25 mg/kg q24 h PO) was started and a clinical cure was observed on April 2009, but the low level parasitemia (11 amastigotes/mL) persisted. Three months later, the cat died due to road accident and liver and spleen were cultured and tested by PCR. Both organs were found positive by PCR and culture was positive only from the spleen [96].

A rapidly fatal case was reported in 2009 in Portugal [97]. The 4-year-old, DSH, female spayed cat lived in Porto (north of Portugal) but had been living for 1 year in Covilhã (central Portugal). A severe pancytopenia with non-regenerative anemia and red cells autoagglutination was the main clinicopathological abnormality. The cat was FIV and FeLV negative and was treated with a blood transfusion, prednisone and antibiotics (enrofloxacyn). Surprisingly, 10 days after amastigotes were observed in 4% of circulating neutrophils, while anemia was worsened. *Leishmania* infection was confirmed by antibody detection (indirect hemagglutination test) and blood PCR. Euthanasia was required 1 week after a second blood transfusion and allopurinol administration (10 mg/kg q12 h PO) because of clinical deterioration. Amastigotes were found in the spleen and mesenteric lymph nodes [97].

In October 2014, a 6-year-old DSH female cat living in the urban area of Messina (Sicily, Italy) was referred for nodular blepharitis, oral mucocutaneous ulcerative lesions and lymph node enlargement [98]. The cat was moderately anaemic, hyperglobulinaemic and tested negative for FIV and FeLV. Fine needle aspirates of nodules and mucocutaneous lesions showed the presence of numerous macrophages containing amastigotes of *Leishmania*. Parasitic infection was confirmed by antibody detection (IFAT titre: 640) and positive real-time PCR on blood and conjunctival swabs. The cat was treated with allopurinol (20 mg/kg SID PO), which was clinically effective, although the cat remained antibody and DNA positive to *Leishmania*. Allopurinol treatment was stopped after 7 months when a severe itching was reported and in September 2017 the cat relapsed showing the same clinical signs. *Leishmania infantum* was identified by culture, and clinicopathological alterations included anaemia, hyperproteinemia, hypergammaglobulinemia, and hypoalbuminemia. Allopurinol treatment was started again but it was interrupted several times because of the itching side effect observed and the cat died 2 months after the relapse [98].

In Cyprus, FeL was diagnosed in a 6-year-old female neutered DSH cat presented at the end of September 2014 for multiple ulcerated skin nodules on the forelimbs [99]. A cytological and histopathological diagnosis of granulomatous dermatitis with intralesional intracytoplasmic amastigotes consistent with *Leishmania* species was obtained from skin nodule and a high parasite load was found by PCR. Anti-*L. infantum* antibodies were detected by ELISA. The cat was enrolled for an epidemiological investigation and found DNA positive for *Hepatozoon* felis and *Candidatus* Mycoplasma hemominutum, DNA negative for *Mycoplasma haemofelis*, *Candidatus* Mycoplasma turicensis, *B. henselae* and *Ehrlichia/Anaplasma* spp., antibody negative to FIV and antigen negative to FeLV. Only mild hyperproteinemia was found at clinicopathological evaluation and allopurinol (10 mg/kg q12 h PO) was given for 6 months. Clinical signs gradually improved with clinical cure reported by month 2 and until the end of the follow up 30 months after diagnosis. However, DNA and ELISA levels of positivity investigated until month 14 demonstrated that the clinical cure was associated with decreased positivity but a sharp increase in positivity was measured after the end of allopurinol treatment [99].

Pereira et al. [100] have been following up for 4 years an 8-year-old DSH neutered female cat diagnosed with FeL in Lisbon in 2014. First manifestation were palpebral nodules where amastigotes were seen at cytological examination. Anti-*Leishmania* antibodies were detected (IFAT titre: 320) and the cat was FeLV provirus positive, with mild thrombocytopenia, hyperproteinemia and hypergammaglobulinemia. Allopurinol (10 mg/kg q12 h PO) was started and, 2 months later, nodules cured. In December 2017, the cat was presented with bilateral anterior uveitis and the owner reported giving half of the prescribed allopurinol dosage. Antibody titre was 512 and mild anemia, thrombocytopenia, leukopenia with neutropenia and monocytopenia, hypeproteinemia, and hypergammaglobulinemia were found. Allopurinol was given again at 10 mg/kg q12 h and meloxicam and prednisolone eye drops were added. In January 2018, the cat was seen again and uveitis persisted. The cat was very prostrated because of a severe mastitis and amastigotes were seen at cytological evaluation of the fluid obtained with fine needle aspiration from mammary tissue. *Leishmania* DNA was detected by PCR in the mammary fluid and the blood buffy coat. Amplicons were sequenced and *L. donovani* was identified. Meglumine antimoniate (50 mg/kg q24 h by subcutaneous injection [SC]) was added for 30 days and 6 months after the end of antimonial treatment both mastitis and uveitis were cured, antibody titre was decreased and buffy coat PCR was negative [100].

Migliazzo et al. [101] reported in 2015 a case diagnosed in the Liguria region (Italy) in a 5-year-old DSH male cat affected by respiratory signs and nasal discharge, conjunctivits and a diffuse proliferative stomatitis with ulcerations. The cat was FIV antibody and FeLV antigen negative. Histopathology of oral lesions showed a diffuse granulomatous stomatitis with numerous macrophages containing protozoal organisms. Antibody detection (IFAT) was negative for *Toxoplasma* and *Neospora* and positive for *Leishmania* (titre 320). However, the cat was treated just with antibiotics and corticosteroids and was represented because of a clinical deterioration after 7 months. At this time, cytological evaluation of nasal exudate was positive for *Leishmania* amastigotes and the paraffin block of the oral biopsy was examined for *Leishmania* by IHC and real-time PCR techniques and found positive (750 amastigotes/mL). Higher parasite loads were detected in lymph node aspirates (80,000 amastigotes/mL) and conjunctival swabs (350,000 amastigotes/mL). Allopurinol therapy was administered (20 mg/kg BID) and after 6 months, stomatitis and rhinitis had resolved [101].

In 2015, three cases with ocular or periocular manifestations were diagnosed at Vila Real (north of Portugal) in DSH spayed female cats from one household aged respectively 10, 8, and 9 years [102]. Case 1 was referred because of a corneal ulceration but bilateral anterior uveitis and an upper eyelid conjunctival nodule were also observed. The cat was FIV antibody and FeLV antigen negative and the only additional abnormalities were hyperproteinemia and hyperglobulinemia. Diagnosis was confirmed by cytological evaluation of the conjunctival nodule, antibody detection by the direct agglutination test (DAT; titre ≥102,400) and positive blood PCR. Meglumine antimoniate (50 mg/kg q24 h SC for 30 days) and allopurinol (10 mg/kg q24 h PO for 6 months) were given, as well as topical symptomatic treatments. Clinical cure was seen at day 45 but the cat died 6 months after diagnosis because of renal failure. Blepharitis, hepatic lipidosis, glomerulonephritis, bone marrow plasmocytosis with rare *Leishmania* amastigotes in macrophages were seen at necropsy. Polymerase chain reaction was positive from bone marrow, lymph nodes and spleen but negative from liver. Case 2 had corneal white spot (whitish infiltrate and edema of superficial corneal stroma) on the left eye and a severe stomatitis. The cat was found FIV antibody positive and affected by non-regenerative anemia, moderate thrombocytopenia and mild hyperglobulinemia. Extracellular amastigotes were evidenced by cytological evaluation of corneal impression smears. The infection was confirmed by antibody detection (DAT titre: 51,200) and positive blood PCR and the cat was treated as case 1. Two months later a mild improvement was seen, but neovascularization and corneal pigmentation occurred. Case 3 had a nodular dermal lesion on the lower eyelid of the left eye where amastigotes were not seen at cytological evaluation. No abnormality at CBC and biochemical profile were observed, blood PCR was negative but antibody positivity was detected by DAT (titre: 51,200). Allopurinol treatment was started and 1 month later, the eyelid nodule completely disappeared [102].

In 2016, a case was reported from Lisbon (Portugal). A 2-year-old DSH neutered male cat was referred for multifocal nodular dermatitis and ulcerative dermatitis on the four limbs and fever [103]. Skin lesions lasted for a year and were treated with no improvement along the previous 2 weeks with prednisone and antibiotics. Approximately 20–25 small nodules were observed mostly on the outer pinnal surfaces where they had a coalescing pattern, and around the eyelids. The limbs were affected by single large (1–5 cm) ulcerated areas with raised margins at the forearms and the hocks. *Leishmania* infection was confirmed by cytological evaluation (skin nodule), PCR test (skin nodule, EDTA blood, lymph node aspirate) and culture (skin nodule, lymph node aspirate). Additionally, a high anti-*Leishmania* antibody IFAT titre (≥1280) and slightly increased alpha-2- and gammaglobulin levels were found, and the cat was FIV antibody and FeLV antigen negative. Allopurinol (10 mg/kg q12 h) was given for 2 weeks and then meglumine antimoniate (50 mg/kg q24 h SC for 30 days) was added because of limited improvement. On day 45 nodules were cleared but hindlimb ulcerations persisted and surgical debridement and excision of the wound edges were performed, followed by horizontal mattress suture for closure and Robert-Jones bandages for 2 weeks. Removed tissue was examined histologically and a granulomatous inflammation was seen with occasional amastigotes in macrophages. One month after surgery, a complete healing was obtained. Allopurinol was stopped after 18 months when antibody titre was 40 and PCR was negative in EDTA blood and lymph node aspirate. Six months after stopping allopurinol the antibody titre was unchanged and the cat was clinically healthy [103].

In July 2017, a respiratory presentation of FeL was seen in Lisbon (Portugal) in a 12-year-old DSH male neutered cat referred with chronic stertor, inspiratory dyspnea and occasional sneezing [104]. According to the history, there was no nasal discharge or cough and mild hyperproteinemia was the only known clinicopathological abnormality. However, during the previous 5 years, recurrent episodes of pruritic dermatitis occurred and were controlled by topical corticosteroid and endectocide treatments. Rhinoscopy was performed and a multinodular appearance of nasopharyngeal mucosa was seen. Mucosal and lesional biopsies were performed for fungal culture and histopathological evaluation. Fungal culture was negative and a marked infiltrate of macrophages with intracellular amastigotes was seen. The cat was re-evaluated for new nasal mucosal samples and two small eyelid nodules and splenomegaly were detected. Additionally, thoracic radiographs, abdominal ultrasound examination, fine needle aspiration of eyelid nodules, CBC, biochemical profile, urinalysis, anti-FIV and anti-*Leishmania* antibody detection, FeLV DNA and RNA detection by blood PCR were obtained. Free and intra-macrophagic amastigotes were observed at cytological evaluation of skin nodules. Nasal nodules were *Leishmania*-PCR positive, and anti- *Leishmania* antibodies were detected (IFAT titre: 320). The cat was FIV and FeLV negative and the only clinicopathological abnormality were hyperproteinemia, hypergammaglobulinemia and mild proteinuria. Allopurinol (10 mg/kg q12 h PO) was given but had to be stopped after 5 days because of an adverse skin reaction. Meglumine antimoniate (50 mg/kg q24 h SC) was given but was stopped after 25 days of treatment because of azotemia and, during antimonial therapy, uveitis was also seen. After that, dietary supplementation with nucleotide and active hexose-correlated compounds (N-AHCC) and benazepril were administered but a relapse of rhinitis was clinically observed 4 months later. At this point miltefosine (2 mg/kg q24 h PO) was added for 28 days and respiratory signs rapidly improved but azotemia occurred on day 21 and required fluid and supportive therapy. The cat had no clinical recurrence during a 4 month follow up period while fluid therapy, renal diet and he supplementation with N-AHCC were administered [104]. 

An additional atypical respiratory form of FeL was reported in Barcelona (Spain) in 2018 [105]. A 5-year-old castrated male DSH cat was referred with a history of chronic nasal discharge and nasal stridor, moderate eosinophilia and mild hyperglobulinemia. Computed tomography (CT) of the head was performed and an ill-defined mass of soft tissue attenuation was seen. The mass occupied the right nasal cavity and extended to the left nasal cavity and the rostral cranial cavity occluding the nasopharynx. Histopathology of the rhinoscopically biopsied lesions revealed a diffuse intense mixed inflammatory infiltrate within the submucosa, mainly consisting of macrophages and scarce multinucleated giant cells, with multiple intracytoplasmic parasitic forms. The IHC evaluation definitively confirmed *Leishmania* spp. infection as the causative etiology of the destructive granulomatous rhinitis. The cat was at first treated with non-steroidal anti-inflammatory drugs (meloxicam, 0.1 mg/kg q24 h PO) and systemic antibiotic (doxycycline, 10 mg/kg q24 h PO) based on positive culture for coagulase negative *Staphylococcus* spp. Following the histopathological diagnosis, a 6 month allopurinol treatment (10 mg/kg q12 h) was added and clinical signs progressively resolved. At 4 month recheck, hyperglobulinemia and antibody positivity still persisted but CT exam demonstrated the complete resolution of the nasal and intracranial mass lesions [105].

Recently, Fernandez-Gallego et al. [106] published a retrospective multicentric study reporting 16 cases diagnosed in Spain (Barcelona, Mallorca, and Valencia) after 2000, but one of them had been previously published in 2005 and it is available in MEDLINE bibliographic database [107]. Inclusion criteria included detection of *Leishmania* amastigotes and/or DNA in cytological or histological samples and/or a high antibody titre, and compatible clinical findings and pathological abnormalities but the clinical management of these cats and the duration of follow up were extremely variable [106]. Fourteen cats were DSH and two were Siamese breed. Age range was 3–21 years, seven cats were males and there were eight females (in 1 case sex was not recorded). Skin lesions were the most common clinical finding, found in 75% of cats and they were associated with non-cutaneous abnormalities in 58% of these cats. Skin lesions consisted more frequently in nodular or ulcerative dermatitis, distributed on the face or distal limbs and less frequently on paws or over the trunk. Exfoliative dermatitis or alopecia were rarely seen. A systemic involvement was detected in 69% of cats. Anorexia and ocular disease (panuveitis and conjunctivitis representing the most frequent lesions) were the most common non-dermatological findings (37.5% each), followed by lethargy, weight loss and renal abnormality (31% each). Oral disease (stomatitis and/or glossitis) or generalized lymphadenopathy were seen in 25% of cats, and hepatomegaly or fever in 12.5%. Single cases of neurologic signs compatible with diffuse central nervous disease, splenomegaly, renomegaly, icterus, vomiting and diarrhea were also found. Investigations for FeLV and FIV infections were performed in 14 cases and five cats were antibody positive to FIV. Potential immunosuppressive conditions were identified in 56% of cats. Most cats (75%) had some clinicopathological abnormalities and the most common were polyclonal gammopathy (86%), anemia (37.5%) and proteinuria (25%). Interestingly, in half of the cases, amastigotes were seen at cytological evaluation of skin or mucosal lesions or of smears from lymph nodes, bone marrow, spleen or liver. Similarly, histopathology confirmed diagnosis from skin, spleen or enucleated eye. *Leishmania* DNA was investigated in 11 samples from skin lesions (3 samples), spleen (2 samples), bone marrow (1 sample), EDTA blood (5 samples) by different PCR techniques and it was always found positive. Antibody positivity (ELISA or IFAT tests) was detected in 91% of 11 tested cats. The course of the disease and the follow up varied. No specific treatment was administered to four cats which were euthanized soon after diagnosis (3 cats) or 24 months later (1 cat). The other cats were treated and allopurinol was administered to 11 cats (in most cases 10 mg/kg q12 h) with variable duration of treatments and outcome (from clinical cure to no response). Allopurinol was associated with meglumine antimoniate in five cats (50 mg/kg q24 h SC for 30 days) or miltefosine in one cat (2 mg/kg q24 h PO for 28 days) or with surgery (nodulectomy, splenectomy, eye enucleation). One cat received only meglumine antimoniate (300 mg q24 h SC) for 4 months with resolution of clinical signs but kidney disease was diagnosed 90 months after the therapy. Kidney disease was seen at diagnosis in five cats or after 5 days of combination therapy with allopurinol and meglumine antimoniate in one cat respectively. The other two cats developed kidney disease respectively 13 and 30 months after the end of therapy [106].

Concerning the prevention of *L. infantum* infection in cats, a longitudinal controlled field study conducted from Spring 2015 to Spring 2016 in the Eolian islands (Sicily, Italy) assessed the efficacy of a 10% imidacloprid/4.5% flumethrin collar against FeL. Based on comparison of yearly crude incidence of *L. infantum* infection by means of seroconversion rates and of blood and conjunctival swab PCR test positivity, the efficacy of the collar in preventing *L. infantum* infection was 75% [65]. In the One Health context, feline strains of *L. donovani* are genetically indistinguishable from those of vector, human, or canine origin [44] and the current most important question concerns infectiousness of cats to sand fly vectors of the parasite. Sand flies are considered opportunistic feeders and feline blood meals are found in *P. perniciosus* sand flies in Spain [108,109] and Portugal [110]. Exposure of cats to sand fly bites was recently demonstrated in Portugal by detection of immunoglobulin (Ig) G antibodies to *P. perniciosus* saliva in cat sera [76]. Interestingly, these antibodies were evidenced in 47.7% of 350 cats investigated in Portugal and sand fly exposed cats had a significantly higher risk of being positive for *Leishmania* infection [76]. However, infectiousness of cats to vector sand flies of *Phlebotomus* spp. was experimentally demonstrated in Europe only from one naturally infected cat submitted to xenodiagnosis [95]. 

##### European Wildcat (*Felis silvestris*)

Scant information about *Leishmania* infection in European wildcats is only available from Spain and Italy, but this endangered felid has a fragmented distribution in other European countries with areas endemic for *L. infantum*.

Between 2001 and 2006, four European wildcats hunted or found dead in northern Spain (Basque Country) were submitted to a post-mortem examination and lesions comparable to those typically found in dogs affected by leishmaniosis were not observed. However, *Leishmania* infection was confirmed by real-time PCR in one out of four (25%) macerates of liver and spleen tissue [34]. This is the first report for this host species.

As part of the wildlife sanitary surveillance program established in Asturias (northern Spain), 149 wild carnivores—including 3 wildcats—that died due to various causes were submitted for necropsy between January 2008 and May 2014. Spleen samples were examined for the detection of *L. infantum* DNA using PCR assay and all the three wildcat specimens were found positive [26].

From 2013 to 2015 *L. infantum* was investigated by PCR in tissue samples of skin, spleen, liver and lymph node collected from 202 dead wild carnivores from southeast Spain. Four wildcats were included in this study and two skin samples were tested, but just one cat (25%) was found positive [27].

A study published in 2020 reported the detection of *L. infantum* DNA in liver and spleen accidental roadkill specimens of a wild cat from Catalonia coastal area using PCR assay [111].

##### Iberian Lynx (*Lynx pardinus*)

Although samples may have been collected before 2001, the only reference to *Leishmania* infection in Iberian lynx comes from Sobrino et al. [25]. One (25%) of four lynxes was found positive by PCR for kDNA in spleen or blood samples opportunistically collected between 1990 and 2007 in central Spain and in the south-western province of Huelva. The positive lynx was from central Spain and had a *Leishmania* PCR-RFLP pattern different from those of dogs [25]. This is the first report for this host species.

##### Barbary Lion (*Panthera leo*)

In 2012, leishmaniosis was diagnosed in a breeding group of Barbary lions kept in captivity at Montpellier Zoological Park [112]. The first case was diagnosed in a 12-year-old female arrived at Montpellier from Morocco in 2003 that became emaciated on January 2010, 6 months after giving birth to a single cub. Colitis with bloody diarrhea, epistaxis, and lameness with ulcers on the four footpads, moderate anemia and leukocytosis, hypercholesterolemia, and hypoalbuminemia were found at clinical examination. Blood PCR was performed for FIV, FeLV, *M. haemofelis*, *Candidatus* M. haemominutum and *L. infantum* and positive results were obtained for the last two pathogens only. The lion was also antibody positive (IFAT titre: 80) and investigations were extended to three other adult lions and the cub. Only one adult clinically healthy female arrived to Montpellier from Morocco at the same time of the *Leishmania*-positive lion was found positive by blood PCR, antibody detection (IFAT titre: 320) and bone marrow cytology and culture. A retrospective evaluation of serum samples of the four adult lions documented antibody positivity of the first case at least since August 2005 and of the second case since November 2008. The symptomatic lion was treated with allopurinol (30 mg/kg q24 h for 3 months) and marbofloxacyn (2 mg/kg q24 h for 28 days). After 3 months of treatment clinical cure was observed and the lion was negative at IFAT, blood PCR and culture. Intermittent (1 week per month) administration of allopurinol was continued. Other wild carnivores were tested in the same zoological park and were found antibody negative [112]. This is the first report for this host species.

##### Tiger (*Panthera tigris*)

In February 2019, DNA of *L. infantum* was detected in a skin biopsy punched from a non-healing large skin ulceration on the trunk of a tiger born and raised in a zoological park in the Apulia region (South Italy) [113]. However, histological and immunohistochemical examination carried out did not evidenced amastigotes and a chronic ulcerative dermatitis with vasculitis/perivasculitis and plasma cellular infiltrate was seen [114]. From March to June 2019 this tiger and additional 19 individuals born and raised in the same open enclosure (age range: 6 months–11 years) were examined and tested for *L. infantum.* Investigations included anti-*L. infantum* antibodies by IFAT, parasite DNA by quantitative PCR from lymph nodes, whole blood, skin punch biopsy, conjunctival, nasal and oral swabs. Lymph node aspirates were also cultured and evaluated by cytological examination and they were found negative. Antibody positivity was 45% with a cut-off dilution of 1:40 (titre range: 40–640) and 25% of titres tested positive by PCR (in 1 or multiple samples apart from conjunctival swabs). All PCR positive tigers were antibody positive and overall prevalence was 25% [113]. All the examined tigers were PCR negative for FIV and FeLV infections and when complete blood count, biochemical, serum protein electrophoresis and urinalysis values of *L. infantum* positive and negative tigers were compared, a significant difference was found about total proteins, globulins, gamma-globulins and haptoglobin [113,114]. This means that despite the lack of manifestations observed at physical examination, *L. infantum* infection was associated in tigers with dysprotidemia due to increased phase acute proteins and gamma-globulins. This is the first report for this host species.

Tigers were probably infected by sand flies, as between May and November 2019, *P. perniciosus* was the most abundant sand fly species collected in the tigers’ paddock and, interestingly, approximately 33% of examined females were positive for tiger DNA and 5.3% for *L. infantum* DNA [113]. 

#### 3.2.3. Family Herpestidae

##### Egyptian Mongoose (*Herpestes ichneumon*)

Five (4.7%) of 106 Egyptian mongooses originated from accidental road trampling or legal actions for predator control, in continental Portugal, were positive for *Leishmania* kDNA by PCR on spleen samples. Positive animals were from the geographical districts of Viseu (central; *n* = 1), Portalegre (eastern; *n* = 1) and Beja (southern Portugal; *n* = 3); with sequence analysis revealing *L. infantum* [115].

#### 3.2.4. Family Mustelidae

##### Eurasian Otter (*Lutra lutra*)

*Leishmania* DNA was detected in the spleen of seven (70%) of 10 wild otters from Asturias, north-western Spain, submitted for necropsy from January 2008 to May 2014 [26]. This is the first report for this host species.

In August 2019, a 4-year-old male Eurasian otter, housed at a wildlife park in Murcia, south-eastern Spain, presented bilateral epistaxis, anorexia, apathy and weight loss. A complete blood cell count (CBC) and serum biochemical profile revealed decreases in platelets; and hyperproteinemia, hyperglobulinemia, decrease in paraoxonase-1, increases in haptoglobin and ferritin, and proteinuria. Bilateral nephropathy with hydronephrosis, mesenteric lymphadenomegaly and ascites were also observed. Urinalysis showed proteinuria and decreased osmolarity and specific gravity. Infection with *L. infantum* was confirmed by cytology (amastigotes in macrophages from a spleen aspirate), serology (specific IgG2 antibodies detected by time-resolved immunofluorometry) and molecular analyses (real-time PCR on blood and bone marrow, and sequencing). Leishmaniosis was diagnosed and allopurinol administered for 3 months, with the animal gaining weight, resolving epistaxis and having the ferritin concentration decreased, but remaining seropositive, at the end of treatment [116].

##### Beech Marten (*Martes foina*)

Three beech (or stone) martens from the community of Extremadura, western Spain, had *Leishmania* kDNA in the hair of their ears or legs, as detected by real-time PCR [23]. This is the first report for this host species.

Six (28.6%) of 21 beech martens from the Spanish Basque Country (the provinces of Biscay, Gipuzkoa and Álava), collected between 2001 and 2006, were detected with *Leishmania* kDNA by real-time PCR on macerates of liver and spleen. A subsequent conventional ITS2-PCR and DNA sequencing confirmed *L. infantum*. None of the animals had lesions suggestive of leishmaniosis at post-mortem examination [34].

Three (66.7%) of nine beech martens from Asturias, north-western Spain, obtained from January 2008 to May 2014 were found positive to *L. infantum* by PCR on spleen samples and DNA sequencing [26].

Three (30%) of 10 beech martens from the region of Murcia, the Valencian community and Andalusia, south-eastern Spain, collected between 2013 and 2015, were found *Leishmania* positive by real-time PCR in tissue samples including skin, spleen or liver. The identification of *L. infantum* was confirmed by DNA sequencing [27].

Three (100%) beech martens from the region of Murcia and the neighbouring province of Alicante, sampled in the period 2008–2017, were positive in spleen, liver or skin by a real-time PCR targeting the ITS2 of *L. infantum*. Analysis of kDNA sequences revealed SNP-derived genotype 2 in martens and also in domestic dogs [28].

Two (50%) of four beech martens collected after accidental road kills in Catalonia, north-eastern Spain, had a qPCR-positive result for *Leishmania* kDNA both on liver and spleen [111].

##### European Pine Marten (*Martes martes*)

Three (30%) of 10 pine martens from the Spanish Basque Country (the provinces of Biscay, Gipuzkoa and Álava), collected between 2001 and 2006, were detected with *Leishmania* kDNA by real-time PCR in macerates of liver and spleen. *Leishmania infantum* was confirmed by a subsequent conventional ITS2 PCR and DNA sequencing. None of the animals had lesions suggestive of leishmaniosis at post-mortem examination [34].

On the island of Mallorca, Spain, nine (39.1%) of 23 pine martens analysed during the period October 2008–October 2009 were found positive by PCR for *Leishmania* kDNA in blood of live and spleen of road-killed animals. Infected pine martens were detected in all seasons, but with a non-statistically significant difference between the warm season (April–September) and autumn to winter (October–March) prevalence values (55.6% [5/9] versus 27.3% [3/11], respectively). No external lesions compatible with leishmaniosis were observed. The existence of a sylvatic cycle could be supported by the fact that no RFLP pattern was shared between pine martens (*n* = 3) and domestic carnivores (*n* = 37) [44].

Spleen samples from five (62.5%) of eight pine martens from Asturias, north-western Spain, obtained between January 2008 and May 2014, were found infected with *L. infantum* as detected by PCR and DNA sequencing [26].

##### European Badger (*Meles meles*)

*Leishmania* kDNA was detected by real-time PCR in macerates of liver and spleen from 14 (26.4%) of 53 European (or Eurasian) badgers collected between 2001 and 2006 in the Spanish Basque Country (the provinces of Biscay, Gipuzkoa and Álava). Badgers were most prevalent in the southernmost province of the Basque Country (Álava) in areas dominated by arable land. *Leishmania infantum* was confirmed by a subsequent conventional ITS2 PCR and DNA sequencing, showing high sequence homogeneity with ITS2 sequences of *L. infantum* from dogs and humans from southern Spain. None of the animals had lesions suggestive of leishmaniosis at post-mortem examination [34].

Twenty-four (53.3%) of 45 European badgers collected after road kills in the region of Piedmont, north-western Italy, between 2009 and 2017, were positive for *Leishmania* by a conventional PCR on spleen samples. DNA sequences showed 100% identity with *L. infantum* [29].

##### European Mink (*Mustela lutreola*)

One (50%) of two European minks (syn. Russian or Eurasian minks) from the Spanish Basque Country was detected with *Leishmania* kDNA by real-time PCR in macerates of liver and spleen. A subsequent conventional ITS2 PCR and DNA sequencing confirmed *L. infantum*. The animal had no lesions suggestive of leishmaniosis at post-mortem examination [34]. This is the first report for this host species.

##### Polecat (*Mustela putorius*)

One (25%) of four polecats from the Spanish Basque Country was detected with *Leishmania* kDNA by real-time PCR in macerates of liver and spleen. A subsequent conventional ITS2 PCR and DNA sequencing confirmed *L. infantum*. The animal had no lesions suggestive of leishmaniosis at post-mortem examination [34]. This is the first report for this host species.

##### Domesticated Ferret (*Mustela putorius* Furo)

In the period February–March 2019, a 4-year-old female pet ferret from Valencia, south-eastern Spain, had an erythematous, oedematous, non-pruritic papular lesion in the right pinna 5 mm in diameter. It had been under treatment with prednisolone and cyclosporine A because of inflammatory bowel disease diagnosed 1 year earlier. Histopathological evaluation revealed severe chronic pygranulomatous dermatitis and intracytoplasmic forms compatible with *Leishmania* amastigotes in macrophages and multinucleated giant cells. Infection with *L. infantum* was confirmed by culture of material aspirated from perilesional excised skin, qPCR targeting kDNA in the paraffin embedded skin biopsy and blood, and immunohistochemistry (IHC). Abnormal clinicopathological results included increased alanine transferase, alkaline phosphatase and serum gamma glutamyl, transferase and polyclonal gammopathy. Antibodies to *Leishmania* were detected by ELISA, IFAT and WB [117]. Thirty days after surgical excision of the original papule, a new lesion was observed at the healing edge of the right ear pinna, which was positive for *Leishmania* in culture. A therapeutic protocol combined allopurinol and meglumine antimoniate during 3 plus 5 weeks, and then allopurinol alone for 4.5 months. A follow-up visit 3 weeks after treatment had been initiated demonstrated clinical response, and the ear pinna lesions had almost disappeared, but culture was again positive for *Leishmania*. Six months after starting with allopurinol, xanthine crystalluria was observed in urine sediment with no other alterations detected by urianalysis. Nine months after the diagnosis of leishmaniosis had been made, the ferret was examined because of severe weight loss, apathy, diarrhoea and dyspnoea with tachypnea. Oocysts, antigens and DNA of *Cryptosporidium* spp. were detected in faecal samples from the patient, which cohabited with two other ferrets diagnosed with cryptosporidiosis. Allopurinol was re-added to therapy and at the last reported evaluation the patient’s general clinical status was stable [118].

##### Stoat (*Mustela erminea*)

In Asturias, north-western Spain, two stoats (syn. short-tailed weasels) were found negative to *L. infantum* by PCR on spleen samples [26].

##### Least Weasel (*Mustela nivalis*)

In Mallorca, Spain, two least weasels analysed in the period 2008–2009 were found negative for *Leishmania* kDNA by PCR (Millán et al., 2011). In the Spanish Basque Country, none of two least weasels were detected with *Leishmania* kDNA by real-time PCR on macerates of liver and spleen [34].

##### American Mink (*Neovison vison*)

In a mink farm in Greece, in April 2015, three (21.4%) of 14 kits affected by hemorrhagic pneumonia were positive for *L. infantum* DNA in brain, liver or spleen detected by ITS1-nPCR. The severity of the pneumonic disease, which was due to *Pseudomonas aerugionosa*, might be attributed to immunosuppression caused by *L. infantum* [119].

Forty (20%) of 200 American minks slaughtered or euthanized in six farms located in the regional unit of Kastoria, Western Macedonia, northern Greece, had serum IgG antibodies to *Leishmania* as detected by ELISA. The sampling period (October–December 2014) and the animal age (7 months) provided that all minks had lived one period of sand fly activity Infection was confirmed in two (2.1%) of 95 animals by ITS1-PCR on spleen samples. All molecularly positive samples were from seropositive minks and no external lesions, splenomegaly or hepatomegaly were observed in any of the animals. Sex was statistically associated with seropositivity, with female minks being almost twice more frequently infected than males (28.2% versus 17.4%). Infection was also statistically associated with the body condition of minks, as seropositivity was much higher in animals with bad (55.6%) than with good (17%) body condition [120].

One American vison collected after an accidental road kill in Catalonia, north-eastern Spain, had a qPCR-positive result for *Leishmania* kDNA on liver and spleen [111].

#### 3.2.5. Family Phocidae

##### Mediterranean Monk Seal (*Monachus monachus*)

A female Mediterranean monk seal, aged around 20 years, found in January 2005 on the coast at Bodrum, southwestern Turkey, was found to be infected with *Leishmania* and parapoxvirus. Pathological findings included a deep ulcer on the side of the head, ulcers on the gingival and inner aspect of the lower lip, enlarged lymph nodes and tonsils, and respiratory lesions. Leishmaniosis was diagnosed histologically and immunohistochemically by the presence of amastigotes in macrophages and reticular cells of lymph nodes, spleen and tonsils [121].

#### 3.2.6. Family Ursidae

##### Brown Bear (*Ursus arctos*)

One bear from a zoological park in Murcia, sampled in the period 2008–2017, was positive in skin by a real-time PCR targeting the ITS2 of *L. infantum*. Analysis of kDNA sequences revealed SNP-derived genotype 2 in that brown bear, as well as in domestic dogs [28]. This is the first report for this host species.

#### 3.2.7. Family Viverridae

##### Common Genet (*Genetta genetta*)

Four (40%) of 10 common genets from the Spanish Basque Country, collected between 2001 and 2006, were detected with *Leishmania* kDNA by real-time PCR in macerates of liver and spleen. A subsequent conventional ITS2-PCR and DNA sequencing confirmed *L. infantum*. None of the animals had lesions suggestive of leishmaniosis at post-mortem examination [34].

On the island of Mallorca, Spain, one (10%) of 10 common genets analysed during the period October 2008–October 2009 were found positive by PCR for *Leishmania* kDNA in blood of live and spleen of road-killed animals. No external lesions compatible with leishmaniosis were observed [44].

*Leishmania infantum* DNA was detected in two (9.1%) of 22 wild genets captured from 2011 to 2013 in the natural parks of Collserola and Sant Llorenç del Munt, near Barcelona, north-eastern Spain. The two infected genets, both females, were found positive by means of real-time PCR in blood, but did not show any external clinical signs compatible with leishmaniosis on physical examination. When compared to the negative females, one infected female had increased concentrations of gamma-globulins and cholesterol; and the other one presented higher creatinine, bilirubin, and chloride levels. These observed alterations have been observed in dogs suffering from leishmaniosis [122].

Three (75%) of four common genets from Asturias, north-western Spain, obtained from January 2008 to May 2014 were found positive to *L. infantum* by PCR and DNA sequencing in spleen samples [26].

One common genet from south-eastern Spain, obtained between 2013 and 2015, was found *L. infantum* positive by real-time PCR in skin samples and DNA sequencing [27].

One genet from the region of Murcia or the neighbouring province of Alicante, sampled in the period 2008–2017, was positive in spleen, liver or skin by a real-time PCR targeting the ITS2 of *L. infantum*. Analysis of kDNA sequences revealed SNP-derived genotype 2 in that genet and also in domestic dogs [28].

#### 3.2.8. Family Procyonidae

##### Raccoon (*Procyon lotor*)

Two raccoons from south-eastern Spain, obtained between 2013 and 2015, were found negative for *L. infantum* by real-time PCR in skin and other tissue samples of spleen, liver or lymph node [27].

### 3.3. Order Chiroptera

#### 3.3.1. Family Vespertilionidae

##### Common Pipistrelle (*Pipistrellus pipistrellus*)

Sixteen (59.3%) of 27 common pipistrelles from the community of Madrid, central Spain, were found positive to *Leishmania* DNA by a conventional PCR on spleen, hair and blood clot samples. Only two animals tested positive in all three sample types. Spleen was the sample yielding the highest number of positive results (*n* = 14; 51.9%), while seven (25.9%) samples of hair and six (22.2%) samples of blood clots were positive. The obtained DNA sequences had 98–100% homology with *L*. *infantum* [123]. This is the first report for this host species.

#### 3.3.2. Family Miniopteridae

##### Common Bent-Wing Bat (*Miniopterus schreibersii*)

None (0.0%) of 35 common bent-wing bats (syn. Schreiber’s long-fingered bats or Schreiber’s bats) from Catalonia, north-eastern Spain, had *Leishmania* DNA in their blood as assessed by a PCR targeting kDNA [124].

### 3.4. Order Diprotodontia

#### 3.4.1. Family Macropodidae

##### Bennett’s Wallaby (*Macropus rufogriseus*)

An autochthonous case of visceral leishmaniosis was described in a captive 2-year-old female Bennett’s wallaby (syn. red-necked wallaby) found dead in its cage in a Madrid zoo, central Spain. No previous clinical signs had been observed. At necropsy, external examination provided normal results. The spleen was dark brown to black, enlarged, with numerous granulomas (0.5–2 cm in diameter). The liver was also dark brown and enlarged, containing white spots and small granulomas (<0.5 cm in diameter). Pulmonary edema and generalized hyperemia were also observed. Macrophages with amastigote forms were found in the spleen and liver by histopathological analysis and confirmed by immunohistochemical detection with antibodies specific for *L. infantum* [125]. This is the first report for this host species.

In another study carried out between June 2011 and September 2012 in the same zoo, *Leishmania* infection was detected in two live animals by PCR (peripheral blood) and serology, and by PCR (lymph node aspirate). Post-mortem PCR analysis of liver, lymph node, skin, kidney, spleen or lung revealed *Leishmania* in four wallabies that died over the study period, including the two animals previously found positive. One of the dead wallabies had severe anaemia, but was once negative for *Leishmania* by PCR and serology while it was alive. Taking into account that DNA sequencing identified *L. infantum*, which is endemic in Spain, and that the four cases were born in Madrid, infections seem to have been locally acquired. Furthermore, detection of the phlebotomine sand fly *P. perniciosus* in the zoo suggests local transmission [126].

### 3.5. Order Eulipotyphla

#### 3.5.1. Family Erinaceidae

##### European Hedgehog (*Erinaceus europaeus*)

One hedgehog from the community of Extremadura, western Spain, had *Leishmania* kDNA in an ear hair sample, as detected by real-time PCR [23]. This is the first report for this host species.

Twenty-two (34.4%) of 64 European hedgehogs collected after accidental road kills in Catalonia, north-eastern Spain, had *Leishmania*-positive results by qPCR and/or ELISA. *Leishmania* kDNA was detected in 22 animals: 19 (37.3%) in 51 spleen samples and five (10%) in 50 skin samples. Among 37 hedgehogs assessed by qPCR in two different tissues (spleen and skin), two animals had positive results in both sample types. Six (12.8%) of 47 animals had *Leishmania*-specific antibodies in blood collected on filter paper [111].

#### 3.5.2. Family Soricidae

##### Greater White-Toothed Shrew (*Crocidura russula*)

Two (13.3%) of 15 greater white-toothed shrews captured, in 2011, in the peri-urban area of Barcelona city, north-eastern Spain, were found positive for *L. infantum* DNA by real-time PCR in blood [127].

##### Etruscan Shrew (*Suncus etruscus*)

One Etruscan shrew (syn. Etruscan pygmy shrew or white-toothed pygmy shrew) captured, in 2011, in the peri-urban area of Barcelona city, was found negative for *L. infantum* DNA by real-time PCR in blood [127].

### 3.6. Order Lagomorpha

#### 3.6.1. Family Leporidae

##### European Hare (*Lepus europaeus*)

Nine (64.3%) of 14 European (syn. brown) hares from the Atlantic geographical region of Spain were found positive to *L. infantum* by PCR, DNA sequencing and RFLP. Spleen samples were collected during necropsies performed over carcasses of animals found dead or provided by hunters from 2004 to 2010 [128]. RFLP patterns of *L. infantum* strains revealed no similarities with those previously found in wild carnivores from continental Spain [25].

In northern Greece, 39 (23.5%) of 166 spleen samples collected from hares in the prefectures of Thessaloniki (*n* = 82) and Chalkidiki (*n* = 84), from 2007 to 2011, were found positive by ITS1-PCR for the detection of *Leishmania* DNA. *Leishmania* sequences were confirmed by phylogenetic analysis to belong to the *Leishmania donovani* complex. In addition, the similarity detected between sequences from hares and dogs could be indicative of a possible overlapping of wild and domestic transmission cycles of *Leishmania* spp. in Greece [129].

Thirteen (12.4%) of 105 brown hares from northern and central Greece, collected by hunters and foresters over two consecutive hunting seasons, were found with serum antibodies to *Leishmania* by IFAT. Five (9.6%) of 56 hares had leishmanial DNA in liver samples, as detected by PCR, and sequence analysis of amplicons revealed 99% identity with *L. infantum* sequences. None of tested *Leishmania*-seropositive hares was found to be PCR positive [130].

Six (6.7%) of 90 brown hares from northern Greece were detected with serum IgG antibodies to *Leishmania* by ELISA. The animals were hunted in the regional units of Chalkidiki (*n* = 34), Thessaloniki (*n* = 32), Serres (*n* = 16), Kilkis (*n* = 6), Imathia (*n* = 1) and Kavala (*n* = 1) during three hunting seasons, from September 2014 to February 2016. Infection was confirmed in two (3.6%) of 56 hares by ITS1-PCR on spleen samples. All molecularly positive samples were from seropositive hares and no external lesions, splenomegaly or hepatomegaly were observed in any of the animals [120].

In the province of Pisa, central Italy, two (0.9%) of 222 hares captured with nets in protected areas, from 2011 to 2015, were found positive to antibodies to *Leishmania* spp. by IFAT. The two seropositive hares lived in plain areas, were males and juveniles [131].

Five (9.8%) of 51 apparently healthy wild brown hares captured with nets in the province of Pisa, in the period 2016–2017 winter, were positive for *Leishmania* spp. DNA by ITS1-PCR on blood [132]. Sequence analysis revealed in all cases 99% homology with *L. infantum*, and with the *L. donovani* complex reported in Greece by Tsokana et al. [129].

##### Iberian Hare (*Lepus granatensis*)

Since July 2009, an outbreak of human leishmaniasis developed primarily in the municipality of Fuenlabrada, in the south-west of the Madrid community, central Spain, after the construction of a peri-urban green park (Bosquesur), where a large population of Iberian hares established. By December 2012, a surprising 446 human clinical cases had been recorded (mean incidence of 22.2 cases per 100,000 inhabitants), consisting of two-thirds CL and one-third VL. The mean age was 44 years (range: 2 months to 95 years), 61.0% were males and only 15.2% of the total had immunosuppressive conditions. The agent was identified as *L. infantum* and high densities of *P. perniciosus* were found. No increase in seroprevalence of *Leishmania* infection was detected during that period in dogs [133]. Between 2010 and October 2016, a total of 691 human cases of leishmaniasis was reported [109].

An entomological study was conducted in September and October 2011 in order to analyse by PCR the blood feeding preferences of sand flies captured very close to the peri-urban park. Analysis of 10 *P. perniciosus* sand flies showed a high preference for hares (*n* = 6), followed by humans (*n* = 3) and cats (*n* = 1), suggesting an association between *P. perniciosus*, hares and humans in the focus. In addition, 79 (58.5%) of 135 *P. perniciosus* were positive for *L. infantum* by PCR [108].

Four (57.1%) of seven apparently healthy but rK39-seropositive Iberian hares from Fuenlabrada were found to be infectious to a mean of 4.7% (range: 0–16%) of *P. perniciosus*. This is the first evidence of the transmission of *Leishmania* from naturally infected hares to a competent vector, and that they may play a role as reservoirs in Europe. Molecular characterisation of isolates obtained from sand flies infected after xenodiagnosis demonstrated that the hares were infected with *L. infantum* identical to ITS type Lombardi [134]. This ITS type was found in isolates from human cases of CL or VL associated with the outbreak [135].

One (20.0%) of five Iberian hares from the northern plateau geographical region, three (60.0%) of five from the north-east, six (60%) of 10 from the centre, 21 (38.8%) of 54 from the southern plateau, and one (50%) of two from the Guadalquivir river valley, Spain, were found positive to *L. infantum* by PCR, DNA sequencing and RFLP. Spleen samples were collected during necropsies performed over carcasses of animals found dead or provided by hunters from 2004 to 2010. The finding of positive hares in the five geographical regions surveyed suggests that *L. infantum* is widely spread in Spanish hare populations [128]. RFLP patterns of *L. infantum* strains revealed no similarities with those previously found in wild carnivores from continental Spain [25].

Sixty-three (74.1%) of 85 hares captured within the area of the Madrid outbreak were found seropositive by IFAT to *L. infantum* promastigotes. The percentage of high titres of antibodies (800–3200) in hares (12.9%) was higher than in rabbits (5.6%) [82].

In 69 Iberian hares captured using nets, in the period September–November 2013, in a north-western area and a north-eastern area of the Madrid region, combined positivity by IFAT (cut-off titre: 25) and n-PCR on skin samples was 31.9% (*n* = 22). No gross pathology compatible with disease was observed [136]. A qPCR on skin samples from these 69 plus one hares detected 55.7% (*n* = 39) of positive animals [137].

Seven (100%) Iberian hares from two green areas of the Madrid community (5 caught in 2014 and 2 in 2013) were positive for *L. infantum* DNA by qPCR on spleen and skin samples. Only minimal microscopic lesions, i.e., the presence of macrophages with *Leishmania* amastigotes without any other inflammatory reaction, were revealed through histopathology of lymph nodes of the infected hares [138].

##### European Rabbit (*Oryctolagyus cuniculus*)

Thirty-one (20.7%) of 150 wild rabbits captured on a farm in the province of Granada, south-eastern Spain, from July 2009 to October 2011, were found positive to *L. infantum*. Blood, bone marrow, liver, spleen, heart and skin were analysed by parasitological, serological and/or molecular techniques, with all the six different types of organs found positive in at least one sample. An external visual inspection of the 107 rabbits shot by authorized hunters did not reveal the presence of clinical signs in any of the animals. Cutaneous lesions were found on 10 specimens (23.3%) of 43 rabbits that were captured by ferrets, transferred to the laboratory and more closely inspected. The number of lesions varied from one to four, located on hind legs, ears or nose. In six of those animals, the presence of *L. infantum* was confirmed in the skin. Amastigotes were microscopically detected in the lesions of two specimens, both of which tested PCR positive for *L. infantum*. With the exception of the liver of one these two rabbits, amastigotes were not detected in any other organ. *Leishmania* infection was associated with size (large rabbits were more frequently positive than medium/small ones) and with capture period (more in October 2010 and 2011 than in July and October 2009), but not with sex. Parasite detection by PCR was positively associated with the antibody titre as determined by IFAT. Rabbits were also detected with *Trypanosoma nabiasi*, which seemed to act as a protection factor against leishmanial infection: the presence of *Leishmania* was significantly greater (4.3 times) in rabbits not infected with *Trypanosoma* [139].

In the provinces of Alicante and Valencia, south-eastern Spain, prevalence of *L. infantum* infection by real-time PCR was 0.6% (1/162) in wild rabbits sport-hunted from October 2009 till March 2010. PCR amplification was observed in a spleen sample, but not in skin, which was the only other tissue tested for that rabbit. The remaining 377 tissue samples of lymph node, nasal skin, bone marrow, liver, kidney, heart, pancreas and vulva from the 161 studied rabbits were PCR negative. Seroprevalence of *Leishmania* infection by an ELISA was 0% (0/36) in the wild rabbits; no plasma was available from the PCR-positive animal and it was not analysed for specific antibodies. PCR prevalence was 10.4% (20/193) and 67.4% (29/43) in dogs based on blood and lymphoid tissues, respectively, and 2.0% (8/392) in human blood-donors based on blood samples. Prevalence of antibodies to *Leishmania* was 6.7% (14/208) in dogs and 2.0% (13/657) in humans. All the assessed wild rabbits, dogs and humans had no external lesions compatible with leishmaniosis. Results suggest that wild rabbits may become naturally infected with *L. infantum* in south-eastern Spain, but the risk of developing chronic infections is very low [140].

Eleven (22.9%) of 48 wild rabbits captured in Bosquesur (Madrid), in March and October 2013, were seropositive to *Leishmania* by a commercial rK39 immunochromatographic test. By xenodiagnosis, five (50%) of 10 seropositive rabbits infected a mean of 0.94% (range: 0–2%) *P. perniciosus* sand flies [141], although in a lower proportion than hares [134]. Rabbits were infectious to *P. perniciosus* in March, proving that chronic infection is long-lasting through the non-transmission period, as occurs with hares [134]. Four isolates were successfully grown by culture and *L. infantum* “Lombardi” identified by PCR and DNA sequencing. This is the first evidence on the infectiousness of apparently healthy *L. infantum*-infected wild rabbits to phlebotomine sand flies. In addition, the molecular analysis of *P. perniciosus* caught in Bosquesur from June to September of 2012 (*n* = 70) and 2013 (*n* = 27) showed that blood meal sources were mainly rabbits (*n* = 40, 57.1%, and *n* = 24, 88.9%, respectively), followed by hares (*n* = 28, 40%, and *n* = 2, 7.4%, respectively) [141].

Sixteen (45.7%) of 35 wild rabbits captured within the area of the Madrid outbreak were found seropositive by IFAT to *L. infantum* promastigotes, with most animals (54.3%) having low to medium antibody levels [82].

Fifty-seven (82.6%) of 69 wild rabbits captured for scientific purposes by ferreting during September 2013 in Madrid, central Spain, were positive by IFAT, PCR or culture. IFAT yielded the highest proportion of positive samples (75.4%), with titres ranging from 25 to 800. PCR analysis of skin samples detected 12 (17.4%) positive samples, while *Leishmania* DNA was only found in two (2.9%) spleen samples. *Leishmania infantum* was isolated from nine (13%) of the animals. No statistically significant associations were observed between seropositivity and age (young versus adult) or sex (females versus males). After post-mortem examination, no macroscopic lesions in liver or spleen were observed in all rabbits, a circumstance which suggests a limited clinical impact of the infection in the hosts [142]. In 215 wild rabbits captured also using ferreting, in the period September–November 2013 (including animals from [142], in a north-western and a north-eastern areas of the Madrid region, combined positivity by IFAT (cut-off titre of 25) and n-PCR on skin and spleen was 70.2%. No gross pathology compatible with disease was observed in any case [136]. A qPCR on skin samples from 203 of these wild rabbits detected 79.8% of positive animals [137].

Nineteen (36.5%) of 52 wild rabbits caught by ferret hunting, in the period 2014–2016, in the Madrid peri-urban outbreak areas were found to contain structures immunopositive to *Leishmania* by a direct fluorescence antibody (DFA) assay in at least one of several types of tissues. *Leishmania* antigens were detected by the DFA assay in all tested tissue types, i.e., spleen, lymph node, bone marrow, gastrointestinal tract, liver, pancreas, cardiac muscle, skeletal muscle, lung, kidney, meninges and skin. No macroscopic lesions compatible with leishmaniosis were found in any of the wild rabbits on post-mortem examination. The only histopathological lesions compatible with leishmaniosis were found in lymph nodes and contained small aggregates of macrophages with intracellular granules resembling *Leishmania* amastigotes, but without any other inflammatory cell reaction [138].

In the region of Murcia, south-eastern Spain, 24 (30.0%) of 80 wild rabbits collected between 2013 and 2015 were found positive by real-time PCR in tissue samples including skin, spleen or liver. Rabbits were much more likely to be positive in skin than in the other organs. Lesions compatible with leishmaniosis were not observed in any of the animals. Infection risk was significantly higher in animals from the southern zone of the Murcia region than in those from the south-eastern one [27].

Ten (58.8%) of 17 wild rabbits from the region of Murcia and the neighbouring province of Alicante, sampled in the period 2008–2017, were positive in spleen, liver or skin by a real-time PCR targeting the ITS2 of *L. infantum*. Analysis of kDNA sequences revealed SNP-derived genotype 1 in rabbits, as well as in domestic dogs and humans [28].

In Central Macedonia, northern Greece, 292 domestic rabbits (201 raised in intensive farms and 91 as homestead) were sampled, between May 2014 and June 2016. The samples from these domestic animals were collected after slaughtering in the regional units of Thessaloniki (*n* = 201), Chalkidiki (*n* = 53) and Serres (*n* = 38). In the same time interval, 101 wild rabbits were sampled in the Island of Lemnos, North Aegean Sea, during two hunting seasons (from August to March), with the help of local hunters. *Leishmania*-specific IgG antibodies were detected by ELISA in the serum of 30 (7.6%) of 393 rabbits. The breeding method was statistically associated with infection, with a higher seropositivity of 22% in homestead reared animals and levels of 1% and 5% in wild and rabbits in intensive farms, respectively. In a molecular analysis by ITS1-PCR, parasite DNA was detected in three (2.6%) of 116 rabbit spleen samples. All samples positive by PCR belonged to seropositive animals. No external lesions, splenomegaly or hepatomegaly were observed in any of the rabbits [120].

Three (4.2%) of 71 wild rabbits hunted or found dead in the four provinces of Sicily, from 2015 to 2017, had *L. infantum* kDNA in their spleen (*n* = 2) or skin (*n* = 1) as detected by qPCR. The animals were specifically from the provinces of Enna (*n* = 11), Caltanissetta (*n* = 11), Messina (Aeolian islands; *n* = 32) and Ragusa (*n* = 17), with positives found in Enna and Ragusa. None of the *Leishmania*-infected rabbits showed macroscopic alterations at necropsy [33]. One (2.5%) of 40 rabbits (36 New Zealand and 4 wild European) older than 1 year and bred outdoors in cages in the Messina province was found positive for serum antibodies to *Leishmania* by a rK39 immunochromatographic kit. The seropositive rabbit, a New Zealand 3-year-old white male, was apparently healthy and no amastigotes were detected in lymph node or blood smears [33].

##### Broom Hare (*Lepus castroviejoi*)

Two Broom hares from the Atlantic geographic region of Spain were found negative to *Leishmania* kDNA by PCR on spleen samples collected from 2004 to 2010 [128].

### 3.7. Order Perissodactyla

#### 3.7.1. Family Equidae

##### Horse (*Equus ferus* Caballus)

In December 2000, a 3.5-year-old Bavarian colt, born near the city of Augsburg, state of Bavaria, southern Germany, was noticed with a small dermal nodule close to the right lower eyelid, which was resected under anaesthesia. During the next 2 months, the lump recurred and grew rapidly; the overlying skin was ulcerated. Multiple small nodular dermal lesions developed in the temporal region lateral to the right eye. Thereafter, a 2.5 cm × 4 cm sample of ulcerated skin with an underlying coarse, yellowish-white nodule was also resected. Diagnosis of cutaneous leishmaniosis was based on histopathology, IHC and electron microscopy. The agent was identified as *L. infantum* by PCR and RFLP. *Leishmania*-specific antibodies were not detected in the serum, neither by IFAT nor ELISA. The lesions healed completely within 6 months without any specific treatment. The animal or its dam had never left the area, and autochthonous infection in Germany cannot be excluded [143].

In southern Portugal, a 17-year-old male mixed-breed (Anglo-Lusitanian) horse, living in the Lisbon metropolitan area for more than 6 years, presented a single irregular ulcerated skin lesion of 2.5 × 1 cm on the right metatarsus, which had evolved from small erosion within 2 months. Physical examination did not reveal any other clinical signs. Serological analysis by counterimmunoelectrophoresis (CIE) revealed anti-*Leishmania* antibodies, and leishmanial DNA was detected by real-time PCR on the cutaneous lesion, which healed spontaneously and relapsed after 3 months. Twelve other horses living on the same farm, where three dogs had previously been diagnosed with leishmaniosis, did not present any clinical signs and their CIE results were negative. Seroprevalence for the equine group sample was 7.7% [144].

Nine cases of cutaneous leishmaniosis were described in five horses in Rhineland, western Germany, one horse in Bavaria, two horses in northern Switzerland and one from unknown origin (age range: 3–17 years). All the animals had been living in Germany or Switzerland for more than 1 year, prior to the development of clinical signs. In three of the horses a stay outside of Central Europe was excluded on anamnesis. The initial diagnosis was based on clinical (single or multiple skin nodules on the head, flank, axilla, ear or thorax) and histological findings. Organisms consistent with amastigotes were observed in skin punch biopsies of surgically excised nodules. Amastigotes of *Leishmania* were confirmed in two animals tested by IHC. In four horses (3 from Germany and 1 from Switzerland), a PCR targeting ITS1 and DNA sequencing showed a close phylogenetic relationship to “L. siamensis” (see also European Cattle, under 3.1.1. Family Bovidae). Nodules were surgically removed with no recurrence in some of the animals, while other nodules regressed spontaneously or recurred [14].

In Vila Real, north of Portugal, a 2-year-old male Belgisch Warmbloed Paard horse, which had never travelled outside the country, was found with a 1.5 cm in diameter ulcerated nodular single lesion on the left face. The cutaneous nodule was surgically excised and its histopathological examination revealed an inflammatory reaction comprising macrophages and moderate numbers of amastigote forms, which were evidenced with Giemsa stain. An IHC method further highlighted amastigotes with an intense reaction. A plasma sample had DAT titre of 200, which represents a more than eight-fold increase in comparison with a <25 (negative) result from another plasma sample obtained 13 months earlier. A kDNA sequence was found to have 98% identity with the *L. infantum* closest sequence in GenBank. There was no recurrence after the complete surgical excision [145].

In the Barcelona province, 16 (14.4%) of 111 healthy Andalusian and mixed-breed horses of both sexes and different ages (1–25 years) were positive for serum antibodies to *Leishmania* by a protein A ELISA. Specific lymphocyte proliferation was observed in the blood of 19 (35.2%) of 54 animals. Of the 54 horses for which protein A ELISA and lymphoproliferation were performed, four (7.4%) were positive to both assays [146].

In the Attica region, central Greece, two (0.4%) of 553 horses had IgG antibodies to *Leishmania* as detected by an ELISA. Eighty-five other horses from the Macedonia region, northern Greece, 42 from the Thessaly region, central-northern Greece, and 73 horses from Peloponnese, southern Greece, were found seronegative to *Leishmania*. The overall seroprevalence among Greek horses was 0.3% (2/753). In addition, seven ponies from Peloponnese were also seronegative, as were six mules from Macedonia and seven mules from Thessaly [147].

In the north of Portugal, seven (4%) of 173 horses blood-sampled between November 2008 and July 2010 were found seropositive for *Leishmania* by the DAT. No statistical differences were found among equine categories of gender (female, male and gelding), age (1.5–6, 7–12 and 13–30 years), type of housing (indoors and mixed/outdoors), ability (recreation, farming and sports) and clinical status (apparently healthy and sick) regarding seropositivity to *Leishmania*. Nevertheless, two sick horses, with fever, anorexia and/or intolerance to exercise, were seropositive [148]. 

In the Tuscany region, central Italy, 18 (6.5%) of 277 horses from the rural areas of the Florence, Pisa and Grosseto provinces were seropositive to *L. infantum* by an IFAT in blood samples collected from June to October 2011. However, at a second sampling, from December 2011 to February 2012, all the same horses were found seronegative. Inclusion criteria included: living for more than 2 years in endemic areas; grazing 24 h a day outside; and inhabiting on farms where affected dogs were housed. All animals were apparently healthy and had no skin abnormalities. These results suggest a transient humoral response to *L. infantum* in horses [149].

Ninety-two (13.9%) of 660 apparently healthy horses sampled between 2016 and 2019 in northern and central Italy were found seropositive for antibodies to *Leishmania* by IFAT (cut-off: 40). The animals were from the regions of Lombardy, Piedmont and Emilia Romagna (northern Italy), and Lazio, Tuscany, Marche and Umbria (central Italy), and had a mean age of 9.8 years (599 horses). Seventy samples were positive at a 1:40 dilution, 21 samples at 1:80 and only one sample at 1:160. Eleven (8.8%) of 125 young horses (≤4 years) tested positive, while 58 (16.5%) of 352 animals aged 4–15 years and 21 (17.2%) of 122 animals older than 15 years were seropositive. Age was found to be statistically associated with seropositivity, with the risk of infection increasing (odds ratio [OR] = 1.04) for each 1 year increase in age. Altitude between 200 and 500 m above sea level (a.s.l.) was a risk factor for seropositivity in comparison with altitude above 500 m a.s.l. (OR = 2.68): 18% (31/172) versus 7.6% (11/145), respectively. Meso-dolicomorphic horses (19.2%, 44/229) had a higher risk (OR = 3.44) of being seropositive than the dolicomorphic ones (6.5%, 9/139). A statistically higher seropositivity (OR = 3.34) was obtained in ponies (32.1%, 9/28) compared with other horses (12.2%, 74/597). Following the application of a multivariable model, the highest value of seropositivity was recorded in northern Italy at 200–500 m a.s.l. (22.7%, 22/97) [150].

##### Donkey (*Equus africanus* Asinus)

In Portugal, one (0.5%) of 186 domestic donkeys sampled between February 2015 and February 2016 was found positive for antibodies to *Leishmania* by the DAT. The seropositive animal was an apparently healthy 32-year-old mixed-breed female from the Algarve, southern Portugal, used for work and housed exclusively outdoors, which had been in contact with other animals [151].

Sixty-seven healthy lactating Amiatina jennies, aged 4 to 18 years old, living on a farm in the municipality of Scarlino, Tuscany, were tested for *Leishmania* infection between July 2016 and October 2018. The animals were semi-extensively reared and had lived for at least two transmission seasons in an area where canine *L. infantum* infection has a seroprevalence of approximately 20%. Eleven (36.7%) of 30 jennies had a positive serum antibody response to *Leishmania*, as detected by means of an IFAT (cut-off: 40). In addition, 22 (59.5%) of the other 37 jennies, sampled for blood once (*n* = 15), twice (*n* = 1) or three times (*n* = 21), were also found seropositive at approximately 3, 6 or 10 months after parturition. Overall serological titres ranged from 40 to 320. Blood specimens collected from the 37 jennies scored positive for *Leishmania* DNA by ITS1-PCR in two animals (June 2017), which had weak IFAT titres [152].

### 3.8. Order Primates

#### 3.8.1. Family Hominidae

##### North-West Bornean Orangutan (*Pongo pygmaeus* Pygmaeus)

Clinical leishmaniosis was reported in two orangutans housed at two different centres in the Madrid community, central Spain. The first animal was a 36-year-old male orangutan born in 1981 in The Netherlands (Rhenen) and transferred in 1984 to southern Spain, first to Málaga for 3 years and then to Valencia until it was moved to Madrid (Rainfer) in 2008. The animal was examined in December 2016 due to a clinical picture characterised by severe weight loss and apathy. A CBC and biochemical profile revealed anaemia, neutropenia, hypoalbuminaemia and elevated transaminases; hepatosplenomegaly was also observed by abdominal ultrasonography. In March 2017, clinical signs worsened, mainly with bilateral epistaxis. The second case was a female orangutan of approximately 34-years-old born in the Artis Amsterdam Royal Zoo (The Netherlands) and transferred to Madrid Zoo in 2009. The animal had severe weight loss and apathy in May 2017, but no other apparent clinical signs. A CBC and biochemical profile revealed anaemia, pancytopenia and hypoalbuminaemia. Splenomegaly and pericardial effusion were also observed. In both orangutans, *L. infantum* infection was confirmed by microscopy (amastigotes in bone marrow aspirates), PCR and serology (IFAT). The animals were treated daily with oral miltefosine for 28 days, with the second case receiving a second cycle 4 months after the first one; allopurinol was also given in the second case uninterruptedly for at least 6 months. During follow up, despite good clinical recovery and a stable general condition, the lack of parasitological cure was confirmed molecularly in both blood and bone marrow samples from the two orangutans [153]. This is the first report for this host species.

### 3.9. Order Rodentia

#### 3.9.1. Family Muridae

##### Wood Mouse (*Apodemus sylvaticus*)

In the Granada province, south-eastern Spain, five (20.8%) of 24 wood mice trapped alive and euthanized, between October 2012 and March 2013, had *L. infantum* as detected by PCR-ELISA (ITS1 or kDNA). Two animals were positive in bone marrow, one in spleen, and another two in healthy skin of the ear lobe [154].

In the region of Murcia, south-eastern Spain, three (18.8%) of 16 wood mice collected between 2013 and 2015 were found positive by real-time PCR in tissue samples including skin, spleen or liver. Lesions compatible with leishmaniosis were not observed in any of the animals [27].

One (50%) of two wood mice from the region of Murcia and the neighbouring province of Alicante, sampled in the period 2008–2017, was positive in spleen, liver or skin by a real-time PCR targeting the ITS2 of *L. infantum* [28].

##### House Mouse (*Mus musculus*)

In the municipality of Sesimbra, in the Lisbon metropolitan area, southern Portugal, nine (33.3%) of 27 house mice obtained between May and October 2011 had *Leishmania* kDNA (qPCR) in ear lobe skin samples. The parasitic load ranged from the detection of residual values to almost 400 targeted copies, which are indicative of low parasite burden. Eight (29.6%) animals had amastigote forms in liver smears (Giemsa) and/or spleen histological sections (hematoxylin and eosin), but with no more than three amastigotes per microscopic field. The house mice were captured in the scope of a rodent control program in two private dog shelters. The absence of skin lesions strongly suggests a non-pathogenic course of infection [155].

In the Granada province, south-eastern Spain, two (50.0%) of four house mice trapped alive and euthanised between October 2012 and March 2013 were detected with *L. infantum* by PCR-ELISA (ITS1 or KDNA). One animal was found positive in bone marrow and blood and the other one in the ear lobe [154].

In Central Macedonia (prefectures of Imathia, Kilkis and Thessaloniki), northern Greece, 16 (24.2%) of 66 house mice collected during pest control programs, from December 2013 to January 2016, were found positive by PCR (real-time kDNA or gel-based ITS1). *Leishmania* amastigotes were not detected in any of liver or spleen smears from all the animals, but specific IgG antibodies were detected by ELISA in 13 (50.0%) out of 26 of them. All infected house mice had low parasite burden and, in addition, no external lesions and no splenomegaly or hepatomegaly were observed [156].

Sixteen (88.9%) of 18 synanthropic adult house mice trapped in the municipality of Chauchina, the province of Granada, southern Spain, in the period February–March 2018, had *Leishmania* DNA as detected by ITS1-PCR, kDNA PCR-ELISA or a Multiplex qPCR. The most frequently infected tissue was ear lobe skin (66.7%), followed by bone marrow (44.4%) and spleen (5.6%); in contrast, all liver samples were negative. The parasite burden was estimated to range from 0 to 741 parasites/sample. Mice were aged 1.5–7 months and five animals were males and 13 females, with five of the latter being pregnant at varying stages: three had foetuses, measuring 2–2.5 cm, while the other two had embryos. *Leishmania infantum* DNA was detected in 29.2% (7/24) of the unborn foetuses’ spleen. Infection in foetal samples ranged from 11.1% to 50.0%, with parasite loads of up to 6481 parasites/5 mg of tissue. Mother-to-infant transmission was observed in all females whose gestational stage was sufficiently advanced to allow foetal analysis [157].

##### Algerian Mouse (*Mus spretus*)

One (4.3%) of 23 Algerian mice captured, in 2011, in the peri-urban area of Barcelona city, north-eastern Spain, was found positive by real-time PCR for *L. infantum* DNA [127].

Fifteen (42.9%) of 35 Algerian mice trapped in Catalonia, north-eastern Spain, gave qPCR and/or ELISA results positive for *Leishmania*. Positivity by qPCR was 17.1% in liver (6/35), 14.3% (5/35) in skin and 28.6% (10/35) in spleen samples, with statistically significant differences between spleen and skin and spleen and liver. Three animals were positive for kDNA in all three tissue types sampled for PCR (liver, skin and spleen) and the rest in two or one types. Three (23.1%) of 13 animals had *Leishmania*-specific antibodies in blood collected on filter paper [111].

##### Brown Rat (*Rattus norvegicus*)

In Cyprus, 19 (5.5%) of 344 brown rats collected in the period 2000–2003, in 51 different locations of five prefectures, were positive to IgG antibodies to *L. infantum* as detected by IFAT (cut-off titre: 60) [158].

In the suburban areas of the cities of Athens and Piraeus, central Greece, one (6.3%) of 16 brown rats trapped alive was positive for *L. infantum* by nPCR on spleen and DNA sequencing [159].

In Central Macedonia (prefectures of Chalkidiki, Imathia, Kilkis and Thessaloniki), northern Greece, seven (70.0%) of 10 brown rats collected during pest control programs, from December 2013 to January 2016, were positive by ELISA for IgG antibodies to *Leishmania*. Amastigotes were not detected in any of liver or spleen smears from 18 animals, neither was anyone of them found positive by PCR (real-time kDNA or gel-based ITS1). In addition, no external lesions, splenomegaly or hepatomegaly were observed [156].

In the municipality of Sintra, in the Lisbon metropolitan area, southern Portugal, one (33.3%) of three brown rats obtained between May and October 2011 was positive for amastigotes simultaneously in liver and spleen smears. Positive samples had no more than three amastigote forms per microscopic field. The animals were captured in the scope of a rodent control program in two private dog shelters. Quantitative PCR (kDNA) was negative in ear lobe, liver and spleen samples. The absence of skin lesions strongly suggests a non-pathogenic course of infection [155].

In the community of Extremadura, western Spain, two (33.3%) of six brown rats had *Leishmania* kinetoplastid DNA in the hair of their legs, as detected by real-time PCR [23].

Five (100%) brown rats from the region of Murcia and the neighbouring province of Alicante, sampled in the period 2008–2017, were positive in spleen, liver or skin by a real-time PCR targeting the ITS2 of *L. infantum*. Analysis of kDNA sequences revealed SNP-derived genotype 2 in one rat and also in domestic dogs [28].

Twenty-eight (33.3%) of 84 brown (or Norway) rats trapped in the sewage system of Barcelona, during the winter of the period 2016–2017, were positive for *Leishmania* DNA by qPCR on spleen samples. On the other hand, only one (7.1%) of 14 brown rats trapped in parks was found positive, within the scope of the same study. The estimated number of parasites in the positive samples from spleens (10 mg) varied considerably, ranging from 0.28 to >2200. Neither splenomegaly nor hepatomegaly were evident in the infected animals [160].

##### Black Rat (*Rattus rattus*)

In Cyprus, 17 (11.2%) of 152 black rats collected in the period 2000–2003, in 51 different locations of five prefectures (mainly in residential areas and stables), were positive to IgG antibodies to *L. infantum* as detected by IFAT (cut-off titre: 60) [158].

On the island of Montecristo, Italy, 11 (15.5%) of 71 black rats captured in 2011 and 2012 were positive to *L. infantum* by PCR on spleen. Sequencing confirmed the identification of *L. infantum* DNA. No relevant abnormalities were recorded at necropsy. One (20.0%) of five *Phlebotomus mascitii* sand flies, which were recorded at very low frequencies, tested positive to *L. infantum* PCR. These findings suggest that, in an isolated environment where the introduction of infected hosts can be ruled out in recent years, black rats can maintain *L. infantum* in the absence of dogs or other carnivores [161]. Nevertheless, the presence of wild rabbits on the island should also be taken into account [154].

In the Granada province, south-eastern Spain, three (33.3%) of nine black rats trapped alive and euthanised between October 2012 and March 2013 were detected with *L. infantum* by PCR-ELISA (ITS1 or KDNA). One animal was found positive in blood, another one in bone marrow and the reamining one in the ear lobe. Two other black rats (22.2%) were found with amastigotes in the liver [154].

In Central Macedonia (prefectures of Chalkidiki and Thessaloniki), northern Greece, three (25.0%) of 12 black rats collected during pest control programs, from December 2013 to January 2016, were found positive by PCR (real-time kDNA or gel-based ITS1). *Leishmania* amastigotes were not detected in any of liver or spleen smears from all the animals, but specific IgG antibodies were detected by ELISA in four (50.0%) out of eight of them. All infected black rats had low parasite burden and, in addition, no external lesions, splenomegaly or hepatomegaly were observed [156].

#### 3.9.2. Family Sciuridae

##### Eurasian Red Squirrel (*Sciurus vulgaris*)

Five (20%) of 25 red squirrels collected after accidental road kills in Catalonia, north-eastern Spain, had a qPCR-positive result for *Leishmania* kDNA. Three (12%) of 25 animals were PCR positive in liver and spleen and two (16.7%) of 12 animals were positive in skin [111].

## 4. Birds (Class Aves)

### 4.1. Order Anseriformes

#### 4.1.1. Family Anatidae

##### Greylag Goose (*Anser anser*)

Three (8.8%) of 34 greylag geese from farms located in the Apulia and Basilicata regions, southern Italy, sampled from December 2005 to March 2006, were found seropositive to *L. infantum* by an IFAT (cut-off titre: 30) [162]. This is the first report for this host species.

##### Muscovy Duck (*Cairina moschata*)

In the same southern Italian area as above, seven Muscovy ducks, sampled between December 2005 and March 2006, were all found seronegative to *L. infantum* by IFAT (cut-off titre: 30) [162].

### 4.2. Order Galliformes

#### 4.2.1. Family Phasianidae

##### Common Pheasant (*Phasianus colchicus*)

One (20.0%) of five common pheasants also from the Apulia and Basilicata regions, sampled from December 2005 to March 2006, was found seropositive to *L. infantum* by IFAT (cut-off titre: 30) [162]. This is the first report for this host species.

##### Chicken (*Gallus gallus* Domesticus)

All 73 rural chickens from farms in the Apulia and Basilicata regions, sampled between December 2005 and March 2006, were found seronegative to *L. infantum* by IFAT (cut-off: 30) [162].

#### 4.2.2. Family Numidae

##### Helmeted Guineafowl (*Numida meleagridis*)

Two helmeted guineafowls from southern Italy, sampled from December 2005 to March 2006, were found seronegative to *L. infantum* by IFAT (cut-off titre: 30) [162].

## 5. New Insights into the Future of Epidemiological Aspects of Animal Leishmaniosis in Europe

Apart from infectiousness to sand flies, the reservoir role and the risk to human and animal health, in particular to pets, arises from other important requirements not usually met by wild animals. For instance, in most cases there is no close contact between the wild animals and humans or pets and, as sand flies do not travel long distances [82,163], it is not likely that a lot of transmission will occur. However, as the example of human leishmaniasis outbreak in Madrid has shown, when there is close contact between humans (entering the niche, in this case city parks) and animals (in this case hares), and when the appropriate vectors are present, the high population density of a wild reservoir is able to sustain a human disease outbreak [108,128,134,164].

**Figure 1:**
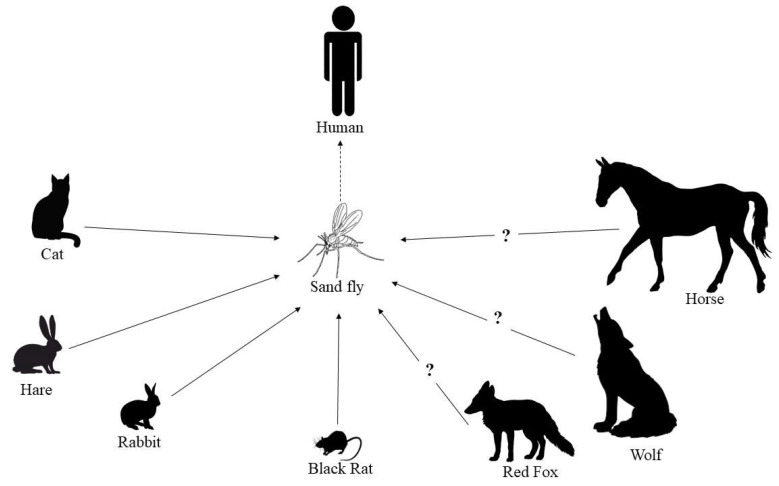
Animals other than dogs proven or suspected (?) as sources of *Leishmania infantum* for *Phlebotomus* spp. and humans in Europe.

The situation might be different when there is more direct contact between humans and infected animals, in particular in case of domestic or farm animals. The present review has shown that there is not much evidence that livestock (cattle, sheep and goats) are infected with *L. infantum*. In contrast, based on the finding in this review, there is reason for concern that equids, and in particular horses (Figure 1), may be a more potential reservoir as increasing numbers of infections, with overt diseases, are being reported [150].

The feline population remains, in addition to dogs, the most important group to be affected by leishmaniosis. As reported in this review, subclinical and chronic feline infections are common in regions endemic for CanL [48,49,50]. Particularly where most dogs are well-protected against sand fly bites and often also by vaccination, cats may play a significant epidemiological role because of their high numbers and the limited use of ectoparasiticides effective in cats against sand fly bites. In fact, in some endemic areas, both pet and unowned cats outnumber dogs [165]. The significance of cats as a reservoir of *Leishmania* spp. and not simply as an accidental host is gaining more and more ground [166]. The recent systematic review by Asfaram et al. [167] further underpinned that cats act as primary and/or secondary reservoir hosts in the transmission of *Leishmania* spp. to humans and also to dogs, through sand flies, at least in endemic foci. This re-emphasises the recommendations for further research to close the knowledge gap in FeL and to gather more evidence-based information on the epidemiology, transmission and management of this disease [40]. Clinical illness due to *L. infantum* in cats is less frequent than in dogs, and though it is currently better recognized by feline practitioners than in the past, it is still underestimated and requires more veterinary attention [40,41,166]. Similarities with CanL exist and cats with *L. infantum*-associated clinical disease have high blood parasitaemia, low to very high antibody levels and hyperglobulinaemia; however, consolidated evidence-based knowledge about the disease associated with *L. infantum* infection in cats is limited, including risk factors, the role of innate and adaptive immune response in the pathogenesis and prognosis, and the more appropriate management of clinical cases.

The red fox is the most abundant wild carnivore in Europe, with relatively high population densities, and can be considered a species that connects the wild, rural and urban environments [168]. Red foxes have displayed high prevalence values and may serve as a secondary reservoir of *L. infantum*, but it is still necessary to elucidate whether they can be infectious to sand flies [11,36]. Several reports are also present in the literature about infected wolves, but the role of this wild species in the epidemiological cycle of *L. infantum* is still unclear (Figure 1).

The geographical spread of animal *Leishmania* infections is mainly confined to the more southern parts of Europe, in particular Greece, Italy, Spain and Portugal, where the Mediterranean climatological conditions for sand flies are probably more favourable. Few cases are reported from more temperate countries such as Germany and Switzerland [94,143]. The Nordic countries have so far not reported cases of animal infections with *Leishmania* in hosts other than dogs. Global warming may eventually change this and it is therefore important to become vigilant [9]. Additionally, travelling and rehoming of cats [94] or movements of wild animals between zoological parks can give rise to occasional clinical cases in non-endemic areas. Nowadays, it is not known whether non-vectorial transmission of *L. infantum* occurs in hosts other than dogs and humans. If this was the case, autochthonous foci could be observed out of endemic regions as is seen in dogs [169].

## 6. Discussion and Conclusions

The present review shows that many mammal species other than humans and dogs can be infected with *Leishmania*, or have at least been in contact with these parasites, as demonstrated by their adaptive immune response. Birds do not seem to be significantly infected by *Leishmania*. The reported observations in mammals range from more or less anecdotal findings, in few cases, to larger epidemiological studies in, for example, cats and hares [41,128,134,167].

Much of the presented evidence is indirect, mostly based on serology (detection of antibodies), but also supported by the detection of specific parasite DNA in many cases. The lack of species-specific antibodies for many of the wild vertebrates in Europe makes it difficult to standardize serological tests and compare data between studies. Current molecular techniques are very sensitive, but a PCR-positive individual does not necessarily mean that it is involved in *Leishmania* transmission, with the same assumption being valid for the other detection methods. Relatively few observations are based on the direct detection of amastigotes in microscopic preparations or successful cultivation, and xenodiagnosis is limited to cats [95] and lagomorphs [108,134]. The infectiousness of experimentally infected black rats to *P. perniciosus* and *Phlebotomus perfiliewi* was demonstrated last century by Gradoni et al. [170].

In particular, the application and interpretation of serology should be approached with caution as cross-reacting antibodies, generated in response to exposure to other infectious agents, may result to false positive results and consequently incorrect incrimination of infection [171,172]. Unfortunately, validated cut-off values or pattern band recognition are not available for antibody detection in non-canine hosts, apart from IFAT and ELISA or WB, respectively, in cats [62]. Importantly, the rapid diagnostic tests largely used in a clinical setting to detect anti-*Leishmania* antibodies in dogs are not validated to test feline sera and must not be used. More in general, the diagnosis of FeL and the management of cats with compatible clinical signs can be challenging for practitioners. In fact, apart from cases when amastigotes are detected in association with tissue (e.g., skin, mucous membranes, eye or lymph node) lesions, it is not easy to confirm a causative role of *L. infantum* in the clinical signs observed, as coinfections and comorbidities can occur [40]. At the same time, treatment of cats with clinical leishmaniosis is still empirically based and off label, using the most common drugs administered to dogs. The infection, however, is not cleared in cats and clinical signs can recur after stopping therapy, as is the case in dogs. Therefore, cats should be carefully monitored under therapy for possible adverse effects (see also Domestic Cat, under 3.2.2. Family Felidae) and after the end of treatments for recurrence of the disease. Moreover, we recommend that owners are asked to sign an informed consent when these drugs are prescribed to cats.

It remains difficult to actually determine whether there is a real infection (and whether it is established or not) or just an exposure, in particular as many “infected” species do not show signs of overt disease. It is, therefore, important that the definition of animal infection is based, at least, on the presence of specific antibodies in combination with the detection of parasite-specific DNA.

In reports where the infecting *Leishmania* spp. could be identified in mammals other than dogs and humans, it was shown that this was, with a few exceptions, in almost all cases *L. infantum*. This is a concern, as this particular species is responsible for potentially fatal human VL [9,173]. In this respect, it would be highly relevant to assess whether the wide range of non-human animal hosts other than dogs are potential reservoirs and actually contribute to transmission of the parasites to either other animals and humans (Figure 1). Xenodiagnosis is the most direct way to determine the infectiousness of infected hosts to the vectors (and subsequently other hosts) and can be used to investigate the dynamics and epidemiology of *Leishmania* transmission [174]. However, there are many logistic hurdles to be taken before xenodiagnosis can be applied to the wild reservoirs.

In geographical areas where *L. infantum* is endemic in dogs and humans, infection and disease caused by this vector-borne agent mut also be considered in non-human animals other than dogs. Surveillance and monitoring programs should assess infection in domestic and wildlife populations and also diagnose diasease at the individual level in clinically suspected animals. Efforts are needed to avoid infections with *Leishmania* in vertebrate species, by means of preventative measures directly applied to animals or their habitats. Further research should investigate the genetic diversity of *L. infantum* that may be shared among domestic and wild animals, to elucidate the epidemiological role of non-human animals other than dogs. These studies could contribute to the development of effective prevention and control strategies with an impact on the *L. infantum* zoonotic infection.

In conclusion, the dynamics of *Leishmania* reservoirs in the European context are complex as there is accumulating evidence that infections with *L. infantum* are not restricted to dogs and, among domestic mammals, cats can be considered the most important additional reservoir species. However, many more mammals can be infected and a potentially huge reservoir may exist. Many infections in wild animals are likely to be accidental, attributed to the opportunistic hematophagous behaviour of sand flies, but they deserve attention, as in some cases they have overt disease or sustain an outbreak of human leishmaniasis.

## Figures and Tables

**Table 1:** Studies reporting percentages of positivity to *Leishmania* tests of cats in Europe between 2001 and 2020.

Country (Area)	Period of Sampling (Month or Season and Year)	No. of Cats (Lifestyle)	Positivity %			% of Non-Healthy Positive Cats (Abnormalities)	Variables Associated with Positivity (Test)	References
			(Immunological Test)	(Parasitological Test: Sample)	Overall ^§^			
Albania (Tirana)	2008–2010	146 (free roaming)	0.7 (IFAT)	0.0 (PCR: blood)	0.7	NA	NA	[51]
Cyprus (all six districts)	March–September 2014	174 (feral, outdoors, indoors)	4.4 (ELISA ^#^)	2.3 (PCR: blood)	5.8 ^#^	Ab = 86.0 (NA) PCR = 100 (NA)	*Hepatozoon* spp., *C*Mt infections (Ov)	[52]
Greece (Thessaloniki)	NA	284 (stray)	3.9 (ELISA)	NA	NA	NA	No	[53]
Greece (Macedonia and Thessaly)	January 2009–September 2011	100 (indoors, outdoors)	11.0 (IFAT IgG and IgM, ELISA)	0.0 (cysm: lynd, skin, bm, conj) 41.0 (PCR: skin, bm, blood, cjsw)	46.0	Ab = 45.5 (skin, ocular, systemic) PCR = 48.8 (skin, ocular, systemic)	Season, FeL compatible signs (PCR)	[48,49]
Greece (Crete, Mykonos, Skopelos and Athens)	Summer 2015	148 (colony, free roaming)	6.1 (IFAT)	6.1 (PCR: blood)	6.1	NA	NA	[54]
Greece (Thessaly)	NA	150 (stray, NA^)	NA	13.3 (PCR: blood)	NA	NA	No	[55]
Greece and Italy	NA	269 (indoors, outdoors)	3.0 (IFAT)	NA	NA	NA	Cohabitation with dogs	[56]
Italy (Abruzzo)	September 2002–March 2004	203 (stray, indoors, outdoors)	16.3 (IFAT)	45.5 (PCR: blood) ^a^ 100 (PCR: lynd) ^a^	NA	66.4 (heterogeneous)	NA	[50]
Italy (Greater Milan)	January 2008–January 2010	233 (colony)	25.3 (IFAT)	0.0 (PCR: blood)	25.3	79.7 (heterogeneous)	Neutrophilia, FIV positivity (Ab)	[57]
Italy (Milan)	June–December 2014	90 (stray)	12.2 (IFAT)	1.1 (PCR: blood) 1.1 (PCR: lynd) 0.0 (PCR: cjsw)	12.2	100 (lymphadenomegaly, stomatitis, skin)	FCoV positivity	[58]
Italy (Milan City, northern Italy)	June 2016–December 2018	117	4.9 (IFAT)	4.3 (PCR: lynd) 0 (PCR: blood) 0 (PCR: cjsw)	8.6	NA	Hypergammaglobulinemia	[59]
Italy (Umbria, Tuscany and Marche)	2010–2016	286 (cattery, colony)	10.8 (IFAT)	15.73 (PCR: cjsw) 0.0 (PCR: bc)	21.7	0.0	Geographical area (Ab, PCR) Age class (Ab) Lifestyle (Ov)	[60]
Italy (Sardinia)	October 2011–January 2013	90 (outdoors, indoors)	10.0 (IFAT)	5.5 (PCR: bc)	14.4	38.5 (heterogeneous)	No	[61]
Italy (Sicily and Calabria)	March 2012–January 2013	76 (outdoors, indoors, stray)	30.3 (IFAT, ELISA, WB)	NA	NA	NA (heterogeneous)	NA	[62]
Italy (Sicily and Calabria)	March 2012–January 2013	197 cats (outdoors, indoors, stray)	9.6 (IFAT)	6.6 (PCR: cjsw, osw, blood, lynd, urine)	14.7	NA	No	[63]
Italy (Sicily)	March 2016–April 2017	66 (indoors, outdoors, stray)	21.0 (IFAT, ELISA) 17.0 (LSA-IFNγ ^&^)	4.0 (PCR: blood)	36.0	NA	NA	[47]
Italy (Aeolian Islands, Sicily)	January 2015–June 2016	330 (outdoors)	25.7 (IFAT)	2.1 (PCR: blood) 1.8 (PCR: cjsw)	25.7	NA (systemic, skin, ocular)	Age class (Ab)	[64]
Italy (Aeolian Islands, Sicily)	March–April 2016	159 (outdoors)	9.4 (IFAT)	7.5 (PCR: blood) 3.7 (PCR: cjsw)	15.7	NA	NA	[65]
Italy (North, Centre and South)	June 2017–August 2018	2659 (NA)	3.3 (IFAT)	0.8 (PCR: blood)	3.9	NA	Geographical area, age class, neutering status, FIV infection (Ov)	[66]
Italy (Emilia Romagna)	June–November 2017	152 (indoors, outdoors)	11.8 (IFAT)	0.7 (PCR: hair) 0.0 (PCR: blood, cjsw)	12.5	NA	Serum total proteins, β_2_- and γ-globulins (Ov)	[67]
Italy (Emilia Romagna, northern Italy)	February 2018–October 2019	85 (stray)	2.4 (IFAT)	5.9 (PCR: blood)	2.4	NA	NA	[68]
Portugal (Lisbon)	Spring–autumn 2004	23 (stray)	20.0 (IFAT)	30.4 (PCR: blood)	NA	NA	NA	[69]
Portugal (Lisbon)	November 2003–July 2005	180 (stray)	0.6 (IFAT)	NA	NA	NA	NA	[70]
Portugal (Lisbon)	January 2007–August 2008	142 (NA^, stray)	1.3 (IFAT)	20.3 (PCR: blood)	20.4	NA	NA	[71]
Portugal (Northeast)	May 2004–July 2008	316 (indoors, outdoors)	2.8 (ELISA, DAT)	NA	NA	11.1 (NA)	No	[72]
Portugal (North and Centre)	NA	320 (outdoors, indoors)	NA	0.3 (PCR: blood)	NA	0.0	NA	[73]
Portugal (Lisbon and Algarve)	January 2012–August 2013	649 (indoors, outdoors, sheltered)	NA	9.9 (PCR: blood)	NA	NA	Age class, habitat, ectoparasiticide treatment	[74]
Portugal (Algarve)	November 2011–May 2014	271 (stray, NA^)	3.7 (DAT)	NA	NA	NA	No	[75]
Portugal (Centre, Lisbon and Algarve)	April–December 2017	350 (stray, colony, outdoors)	0.9 (IFAT)	6.9 (PCR: blood)	7.4	26.9 (NA)	No	[76]
Portugal (Centre, Lisbon and Algarve)	February 2017–August 2018	465 (stray, colony, NA^)	NA	5.4 (PCR: bc) *	NA	NA	NA	[44]
Spain (South)	February 2003–December 2004	180 (NA^)	28.3 (IFAT)	25.7 (PCR: blood) 42.9 (cysm: bc of seven PCR positive cats	48.3	NA	FeLV (Ab)	[77]
Spain (NA)	2004–2007	2632 (NA)	NA	0.57 (IHC)	NA	NA	NA	[78]
Spain (Madrid)	September 2005–June 2006	233 (indoors, outdoors)	1.3 (IFAT)	0.4 (PCR: blood)	1.7	75 (heterogeneous)	No	[79]
Spain (Madrid)	September 2005–August 2008	680 (indoors, outdoors, stray)	3.7 (IFAT)	0.6 (PCR: blood)	NA	NA	Age class (Ab), FIV (Ab), oral disease (Ab)	[80]
Spain (Madrid)	NA	17 (breeding)	17.6 (IFAT)	NA	NA	NA	NA	[81]
Spain (Madrid)	2011–2012	55	NA	3.7 (PCR: spleen)	NA	NA	NA	[82]
Spain (Madrid)	NA	43 (stray)	9.3 (IFAT)	NA	NA	NA	NA	[83]
Spain (Centre)	Spring 2012 and spring 2013	346 (stray)	3.2 (IFAT)	0.0 (PCR: 57 blood samples)	NA	9.1 (compatible with FeL)	No	[84]
Spain (Centre)	2014–2017	249 (colony)	4.8 (IFAT)	0.0 (PCR: blood, skin)	4.8	16.7 (compatible with FeL)	NA	[85]
Spain (Catalonia and Mallorca island)	NA	445 (mixed)	5.3–6.3 (ELISA)	NA	NA	NA	No	[86]
Spain (Barcelona)	January–Deccember 2006	100 (NA)	NA	3.0 (PCR: blood)	NA	100 (NA)	Age class (PCR)	[87]
Spain (Catalonia)	March 2016–April 2017	113 (indoors, outdoors, stray)	22.0 (IFAT, ELISA) 18.0 (LSA-IFNγ ^&^)	5.0 (PCR: blood)	NA	34 (heterogeneous)	NA	[47]
Spain (Ibiza island)	June–July 2008	105 (shelter outdoors)	13.2 (ELISA)	8.7 (PCR: blood)	15.2	75.0 (heterogeneous)	Clinical signs (Ov) FeLV (Ab, PCR) FeLV, FIV (Ab, PCR)	[88]
Spain (Mallorca island)	June 2008–February 2009	86 (feral)	15.7 (WB)	26.0 (PCR: blood)	25.6	0.0	No	[45]

^a^ PCR tests performed only in 11 antibody positive cats at a different time; ^§^ Immunological and/or parasitological positivity; * *L. donovani* complex (24 cats), *L. major* (2 cats). ^&^ Interferon-γ ex vivo produced by blood cells stimulated with *Leishmania* soluble antigen; ^#^ ELISA test and overall prevalence from 160 cats. Ab: antibody detection; bc: buffy coat; bm: bone marrow; *C*Mt: *Candidatus* Mycoplasma turicensis; cjsw: conjunctival swab; conj: conjunctiva; cysm: cytological smear; DAT: direct agglutination test (cut-off dilution: 1:100); ELISA: enzyme-linked immunosorbent assay (different techniques); FCoV: feline coronavirus; FeL: feline leishmaniosis; FeLV: feline leukaemia virus; FIV: feline immunodeficiency virus; Ig: immunoglobulin; IFAT: immunofluorescence antibody test (cut-off dilution ranging from 1:2 to 1:100); IHC: immunohistochemistry; lynd: lymph node; NA: not assessed/available; NA^: lifestyle not assessed in owned cats admitted to veterinary clinics; Ov: antibody and DNA detection; osw: ocular swabs; PCR: polymerase chain reaction; WB: Western blot.
